# Digital twin and fuzzy framework for supply chain sustainability risk assessment and management in supplier selection

**DOI:** 10.1038/s41598-024-67226-z

**Published:** 2024-07-31

**Authors:** Ibrahim M. Hezam, Ahmed M. Ali, Karam Sallam, Ibrahim A. Hameed, Mohamed Abdel-Basset

**Affiliations:** 1 Department of Statistics and Operations Research, College of Sciences, King Saud University, Riyadh, Saudi Arabia; 2 Faculty of Computers and Informatics, Zagazig University, Zagazig, 44519 Sharqiyah Egypt; 3 Department of Computer Science, University of Sharjah, Sharjah, United Arab Emirates; 4 Department of ICT and Natural Sciences, Norwegian University of Science and Technology (NTNU), 7034 Ålesund, Norway

**Keywords:** Digital twin, Supply chain, Sustainability, Risk assessment, Spherical fuzzy sets, MCDM, CODAS method, Entropy method, Mathematics and computing, Applied mathematics, Computational science, Information technology

## Abstract

Risks in the supply chain can damage many companies and organizations due to sustainability risk factors. This study evaluates the supply chain risk assessment and management and then selects the best supplier in a gas company in Egypt. A comprehensive methodology can use the experts' opinions who use the linguistic variables in the spherical fuzzy numbers (SFNs) to evaluate the criteria and suppliers in this study based on their views. Selecting the best supplier is a complex task due to various criteria related to supply chain risk assessment, such as supply risks, environmental risks, financial risks, regularity risks, political risk, ethical risks, and technology risks and their sub-criteria. This study suggested a new combined model with multi-criteria decision-making (MCDM) under a spherical fuzzy set (SFS) environment to overcome uncertainty and incomplete data in the assessment process. The MCDM methodology has two methods: the Entropy and COmbinative Distance-based Assessment (CODAS) methods. The SFS-Entropy is used to compute supply chain risk assessment and management criteria weights. The SFS-CODAS method is used to rank the supplier. The main results show that supply risks have the highest importance, followed by financial and environmental risks, and ethical risks have the lowest risk importance. The criteria weights were changed under sensitivity analysis to show the stability and validation of the results obtained from the suggested methodology. The comparative analysis is implemented with other MCDM methods named TOPSIS, VIKOR, MARCOS, COPRAS, WASPAS, and MULTIMOORA methods under the SFS environment. This study can help managers and organizations select the best supplier with the lowest sustainability risks.

## Introduction

In long-term planning and delayed tasks, supply chain management is critical for many firms and organizations. There are various components in the supply chain, such as managers, stakeholders, and customers with different positions. Multiple criteria, such as supply, social, and environmental impact performance towards customers and stakeholders in the supply chain. The managers and stakeholders have various needs in sustainable supply chain management, such as economic, social, and environmental^1^. Various studies built a framework to analyze and assess the economic, social, and ecological. The critical step in supply chain management is to evaluate the risks concerned with the sustainability of the supply chain for internal and external factors to improve performance and efficiency. These risks relate to conditions or events that can damage stakeholders' response to the supply chain^2^.

The digital supply chain is integrated as a new solution to the risks and challenges in SC and introduces new solutions and opportunities to overcome these risks. Digital technologies refer to the set of digital applications and platforms such as blockchain, big data, the Internet of Things (IoT), cloud computing, and mobile analytics. These digital technologies convert most businesses to more effective ones and introduce new solutions to obtain the firm's and business aims and profitability^3^. Digital gas industry technology refers to the integration of several technologies, such as cloud services, automation, IoT, and analytics tools and processes. Digital technology in gas firms can increase customer services and experiences. Digital technology in gas firms aims to enhance efficiency, increase productivity, promote sustainability, reduce risks, and provide better clients and experiences^4,5^. Cloud services and big data analytics can aid in optimized production and allow more accurate modeling. The virtual tools and platforms in digital technologies can provide options to track customer usage and explore renewable alternatives. Digitization can aid gas firms by selecting unique energy needs and preferences.

The supply chain sustainability risk assessment and management can aid firms and organizations in enhancing the supply chain process. The supply chain sustainability risk assessment and management can be built into digital twin technology. A digital twin is a critical technology in the future and will be used in various real-world applications. In digital twins, the virtual model of a physical entity is built with digitization^6^. There are multiple benefits of digital twins, such as various data being collected and processed in a virtual way in physical entities. Digital twins can provide a suitable framework for a complete real-time model with a physical layer in a virtual layer^7^. The digital layer can be separated into two layers: the physical layer and the virtual layer:

- The physical layer can be described as an objective entity set. It can deal with various tasks of decision-making coming from multiple information schemes. In this layer, the information coming from various systems can be processed, gathered, and transmitted into a virtual layer. There are two sub-layers in the physical layer: the resource sets and information processing layers. In the information processing layer, the information can be combined and processed. The resource layer can have various resource entities.
- The virtual layer can be named a control or decision layer. The control instruction can return to the physical layer through the virtual and cyber-physical systems. The twin model technology mapped the physical layer with the real-time data collection into a virtual layer. The process of the physical layer can also be mapped into a virtual layer; the virtual system can be formed in the virtual layer. This layer imported the decision and control at the same time.

Digital solutions can provide various features in the supply chain, such as storing, analyzing, and sharing information between multiple firms and organizations, promoting networking, facilitating contact between firms, and sharing services, hence providing better and more effective management in SC^8,9^. Digital Twin in gas firms in SC can reduce supply risks, enhance traceability, increase capacity planning, develop inventory strategies, and suggest various supply sources. Digital Twin is a virtual representation of an item in SC for its real-time monitoring and outlining^10^–^12^. The goal is to integrate the digital twin with SC, enhance the production process, and improve SC's flexibility and capabilities. Digital Twin can provide risk assessment and management in SC, improved efficiency, real-time monitoring and control, and better safety and security^6,13^.

The supplier selection with the supply chain sustainability risk assessment and management is a multi-criteria decision-making problem (MCDM). This problem can be solved by using MCDM methods. The MCDM methods are of interest to the various scientific communities^14^. The MCDM methods have been implemented in various real-world applications^15^ to select the best options from multiple options and conflicting criteria. MCDM can aid decision-makers and experts in evaluation criteria and alternatives. The MCDM methods are used in the supply chain with supplier selection. The issues of MCDM have various uncertainty and vague information. So, the fuzzy MCDM can address and manage uncertainty and ambiguous information in the evaluation process by using fuzzy numbers^16^. There is no solution to decision-making problems accurately due to the opinions of experts and decision-makers. Various solutions have been proposed to solve this problem and deal with and overcome this uncertain information in the evaluation process^16^. The supply chain sustainability risk assessment and management to select the best supplier has various criteria and sub-criteria such as environmental risks, financial risks, supply risks, political risks, regularity risks, technology risks, ethical risks, and social risks so that the MCDM can deal with various criteria with precise and accuracy manner. So, the MCDM methodology positively impacts supply chain sustainability risk assessment and management to supplier selection. The MCDM can be combined with various technologies to obtain accurate and precise results.

The available information can measure the vague and uncertain model. The decision-makers and experts used the crisp numbers to assess the criteria and alternatives in the traditional MCDM. When the decision-makers and experts have clear and complete data, this can lead to uncertainty in the results of the MCDM method. The fuzzy set theory (FS) can handle uncertainty and incomplete data^17^. The spherical fuzzy set (SFS) is an extension of FS and has three membership degrees: membership, non-membership, and hesitant^18^. The main goal of using the SFS is to scale the degree of membership and be hesitant. The SFS can be used to select the level of power. This study proposed an SFS to deal with various vague and uncertain information with the MCDM methodology. The MCDM applied in various applications such as assessment photovoltaic^19^, clean energy supply chain^20^, site selection for underground pumped storage plant^21^, E-learning quality evaluation^22^, waste interaction power plant^23^, 3D seismic analysis^24^, offshore wind power station site selection^25^, E-learning course selection^26^, and air pollution control^27^.

Various studies used the MCDM methodology with the fuzzy framework in the sustainability risk assessment and management of the supply chain. This study used the SFS with the MCDM methodology (SFS-MCDM) to overcome uncertainty and vague information using spherical fuzzy numbers (SFNs). This work used two decision-making methods: the Entropy^28^ and CODAS^29^ methods. These two decision-making methods are implemented with SFS and SFNs. The SFS-Entropy method is used to compute the weights of 8 criteria in this study. The SFS-CODAS method is used to evaluate and rank the suppliers.

The CODAS method was applied in various decision making problems^30^–^32^. The CODAS method is a very novel and robust MCDM method. It has two types of distances, such as Euclidean distance and Taxicab distance. These two distances are used to assess the desirability of alternatives. The CODAS method uses the best features from these two distances^33,34^. The CODAS method can help reduce the chance of instability in the solution and rank and provide more flexibility between distances.

Various studies have applied multiple methodologies in supplier selection problems, such as Pamucar et al.^35^ applied the Fermatean fuzzy framework for green supplier selection. They used the CoCoSo method to rank the alternatives. Pamucar et al.^36^ used fuzzy numbers for supplier selection in healthcare. They used the MACBETH method to rank the other options. ULUTAŞ et al.^37^ used the fuzzy AHP and fuzzy OCRA methods in their methodology for supplier selection. They used fuzzy AHP to compute the criteria weights and fuzzy OCRA method to rank the alternatives. Zakeri et al.^38^ used triangular fuzzy numbers with MCDM methodology for supplier selection. Ulutaş et al.^39^ used a fuzzy methodology for green supplier selection. Khan et al.^40^ used q-Rung orthopair fuzzy hypersoft for green supplier selection.

There are various contributions to this study:

I. The first study used the decision-making framework named the Entropy-CODAS method under a spherical fuzzy set to deal with and overcome the uncertainty in the decision-making to select the best supplier in supply chain sustainability risk assessment and management.
II. This study applied the digital twin technology with three layers such as physical layer, virtual layer, and application layer. The digital twin is used with the MCDM methodology in this study. The digital twin used the data from various sources in the physical world in digital expression. Digital twins are used to reduce SC risks by providing various digital solutions for better performance and effectiveness.
III. The suggested methodology SFS-Entropy-CODAS is used and gives accurate results when there is a lot of uncertainty in the decision-making.
IV. This study can help decision-makers, managers, and organizations select the best supplier under various risks in the supply chain.
V. A sensitivity analysis and comparative analysis are applied in this study to show the stability, reliability, and validation of the obtained results.

The remainder of this study is organized as follows: the literature review is presented in section "Literature review". The description of the main criteria in this study is presented in section "Supply chain sustainability risk assessment and management criteria". The digital twin architecture is presented in section "Digital twin architecture". The material and proposed methodology is presented in section "Materials and methods". The application and discussion of the results are presented in section "Application". The sensitivity and comparative analysis are presented in section "Analysis". The challenges of this study are presented in section "Challenges". The managerial implications of this study are presented in section "Managerial implications". The conclusions are presented in section "Conclusions".

## Literature review

In this literature, the studies related to this work are reviewed and discussed. This section is separated into three parts, in the first part we review the studies related to the supply chain sustainability risk assessment and management, in the second part we review the studies related to the digital twin, finally in the third part, we review the studies related to SFS-Entropy and SFS-CODAS methods.

### Supply chain risk assessment and management

This part presents some works related to supply chain sustainability risk assessment and management. Xu et al.^41^ developed a framework to assess the supply chain risk. They used three primary dimensions in their work: operational risk, social risk, and ecological risk. They used risk assessment analysis to rank the resource allocation. They provided two examples as case studies. Giannakis and Papadopoulos^42^ developed an operational perspective of the supply chain in the risk management process. They developed an analytical procedure for managing supply chain risks and discussed the sustainability supply chain risks. They developed an integrated method for data collection and analysis. They used three main risks: ecological, social, and economic. They applied their methodology in manufacturing companies. They ranked the risks by using the failure mode and effect analysis. The main results show that ecological risks have the highest importance in their study. Gouda and Saranga^43^ addressed sustainability supply chain risk. They adopted various types of risks, such as ecological and social sustainability risks. They collected data from six manufacturing sectors in 21 countries. Their main results show that sustainability can aid in decreasing the risk of the supply chain and the risks in emerging markets. Their results show the suggested work can be practical in OCED countries. Zhang and Song^44^ adopted a framework for evaluating the sustainability risk criteria in blockchain technology with supply chain management. They used three main criteria: ecological, economic, and social. They used the best–worst method for computing the risk criteria importance and used the COCOSO method to rank the alternatives. They used a rough framework to deal with incomplete and vague information. Their results give more insights to managers in blockchain technology with supply chain management by considering the sustainability risks. Valinejad and Rahmani^45^ proposed a comprehensive framework for managing and assessing sustainability risks in the supply chain. They applied their method in a case study of internet service providers. They found the most risks in telecommunication companies are technical and institutional. Their model can be used by anyone in the telecommunication industry, managers, and experts to manage the sustainability supply chain performance for a long time.

### Digital twin

This part reviews the studies on digital twins in the supply chain. Andrea Barni et al.^46^ proposed a framework with the digital twin to assess planned production's sustainability performance. The digital twin is used in their work to gather system data. They used decision-making, multi-entry value networks, and internal validation as case studies. They focused on improving the sustainability performance in value chains. Kamble et al.^47^ presented a systematic review of digital twins and supply chains. They aimed to achieve objective sustainable supply chain performance. They suggested technologies such as IoT, blockchain, and cloud computing in the supply chain with digital twins. Their results show that humans and things must be in a digital twin supply chain. Singh et al.^48^ proposed a framework for analysis of the role of digital twins in the sustainability supply chain. They used the grey causal modeling method for analysis and enhanced the resilience of the food supply chain. They used the data that came from the opinions of experts. They can aid policymakers and food industries. Abideen et al.^49^ proposed a digital twin model to reduce the lead time of the supply chain and manufacturing in production. They used the reinforced machine learning model with digital twins to build the perspective decision support platforms. Dmitry Ivanov^50^ offered a decision-making methodology for using digital twins with the supply chain. They used the digital twin to visualise the physical supply chain in digital form. They used analytic methods for data processing and data modeling. The main results show the digital twin can support and monitor methods in the supply chain.

### SFS-entropy and SFS-CODAS methods

This part will review some studies related to SFS-Entropy and SFS-CODAS methods. These two methods are applied in various decision-making applications and under uncertainty environments such as fuzzy sets, neutrosophic sets, and other extension environments.

Chodha et al.^51^ proposed a MCDM methodology with the Entropy method. They used this framework to choose the industrial robot for arc welding operation. They used the Entropy method to compute the robot arc welding criteria weights. The crisp values were used when rating the requirements in the Entropy method. Zafar et al.^28^ employed an MCDM approach to rank and outline the suitable public blockchain platforms. They used the entropy method to compute the weights of the criteria. They used the Entropy method under crisp numbers in their process. Wang et al.^52^ proposed an MCDM methodology for measuring road transport sustainability. They used the Entropy method to balance goals and conflict criteria and compute the criteria weight of road transportation sustainability performance. They used the Entropy method to decrease the subjective impact of decision-makers and increase objectivity. Shemshadi et al.^53^developed a decision-making model for evaluating and ranking potential suppliers in business firms. They used the Entropy method to compute the weights of suppliers. They used trapezoidal fuzzy numbers to assess the criteria and alternatives in their studies. Li et al.^54^ proposed an MCDM model for machine tool selection. The Entropy method was used in their model to compute the objective weight. They used fuzzy decision-making environments to deal with various vague information. Yadav et al.^55^ proposed a decision-making model with the Entropy method for selecting and ordering dental restorative competitive materials. The weight criteria of various performance-defining attributes are computed by the Entropy method. The Entropy method was used with the crisp number to evaluate the requirements of the experts and decision-makers.

Dominguez et al.^56^ proposed an MCDM method for selecting machines, such as the CODAS method. The CODAS method was used to rank the alternatives. The CODAS method used crisp numbers to evaluate the other options. Gupta et al.^57^ proposed a hybrid decision-making method named the CODAS method for assessing the usability of mHealth to manage and keep track of a person's health. They used the CODAS method to rank the alternatives. The CODAS method was used under a fuzzy framework to deal with incomplete data in their work. Alkan et al.^58^ used the MCDM method to overcome complex real-life applications such as renewable energy. They used the CODAS method to rank renewable energy alternatives based on sustainability development. They used the CODAS method under an interval-valued picture fuzzy environment to deal with uncertainty in evaluating renewable energy. Kumari and Acherjee^29^ proposed a decision-making model for selecting a non-conventional machining process. They used the CODAS method to rank the alternatives. They used crisp numbers to evaluate the options with the CODAS method. Ghoushchi et al.^59^ proposed an MCDM model for clean energy barrier evaluation. They used the CODAS method to rank the alternatives. The CODAS method was integrated with the SFS to reduce the uncertainty.

## Supply chain sustainability risk assessment and management criteria

This section introduces the main criteria of supply chain risk assessment and management. This study used 8 main criteria and 38 sub-criteria. The supply chain sustainability risk assessment and management criteria can aid organizations and managers in ranking and assessing the risk in the supply chain^41,60^. Firstly, experts and decision-makers are invited to select suitable criteria related to Egypt gas firms. The criteria are gathered from literature reviews, interviews, and questionnaires. The chosen criteria are rated for supply chain sustainability risk assessment and management. Then, these criteria are presented to the Egypt gas firm to confirm their suitability. The following criteria are suitable for the Egypt gas firm.

Supply Risks (SRM1): This criterion evaluates the supply chain risks. Supply risks in the supply chain can define the risks of delays and disruptions. Digital SC in gas firms can be vital in efficiently managing SC risks. Digital SC can reduce water pollution risks by introducing a certain amount of gas. (supply chain complexity (SRM1_1), single-source dependencies(SRM1_2), transportation vulnerabilities (SRM1_3), and contingency planning (SRM1_4)).

Financial Risks (SRM2): This factor can be used to evaluate the supplier’s costs and economics. This criterion is related to operational costs and investment costs. financial risk (SRM2) (financial health (SRM2_1), creditworthiness (SRM2_2), bankruptcy risk (SRM2_3), and payment terms (SRM2_4)).

Political Risks (SRM3): This criterion can be used to identify the political risks in the supply chain. Political risk (SRM3) (geopolitical stability (SRM3_1), political unrest (SRM3_2), natural disasters (SRM3_3), and supply chain vulnerabilities in specific regions or countries (SRM3_4)).

Regularity Risks (SRM4): This factor can be used to evaluate the risks of regularity in the supply chain. The risks of the reputational image, product recall, and regularity risk can define legal penalties. Digital SC can keep gas quality by using IoT sensors. regulatory risk (SRM4) (regulatory requirements (SRM4_1), industry standards (SRM4_2), product safety (SRM4_3), and quality standards (SRM4_4)).

Technology Risks (SRM5): This criterion can be used to evaluate the requirements related to innovation and technology in the supply chain. This factor is used to identify risks such as data breaches and potential disruptions. Digital solutions such as IoT and big data analytics can be used to predict climate change and reduce risks in SC. Digital technologies can enable information exchange needed to monitor and manage problems and risks and provide safety and security for gas firms from cyber attacks. Technology risk (SRM5) (technology obsolescence (SRM5_1), cybersecurity vulnerabilities (SRM5_2), data privacy (SRM5_3), and intellectual property protection (SRM5_4)).

Environmental Risk (SRM6): Environmental risks evaluate the risks related to the ecological with the supplier and their operations in the supply chain. Evaluation and assessment of environmental risks can aid in increasing the performance of suppliers by defining a list of environmental risks. Digital SC can reduce environmental risks related to resource depletion, deforestation, and pollution. Environmental risk (SRM6) (greenhouse gas emissions (SRM6_1), water usage (SRM6_2), waste generation (SRM6_3), pollution (SRM6_4), deforestation (SRM6_5), and resource depletion (SRM6_6)).

Social Risks (SRM7): The social risks related to the social impacts in the supply chain, such as working conditions, safety rules, and human rights. Evaluation and assessment of risks in social suppliers have defined risks related to violation of human rights and other risks related to social suppliers’ risks. Digital SC can enhance human rights, equality, and health and safety standards. Social risk (SRM7) (working conditions (SRM7_1), labor rights (SRM7_2), health and safety standards (SRM7_3), diversity (SRM7_4), equality (SRM7_5), and human rights (SRM7_6)).

Ethical risks (SRM8): This factor can be used to evaluate the risks related to ethical and government rules such as transparency and compliance. This factor can aid suppliers in identifying unethical conduct. Digital SC can help detect ethics-related risks, such as transparency, compliance with regulations and standards, conflict minerals, and bribery. Ethical risk (SRM8) (corruption (SRM8_1), bribery (SRM8_2), conflict minerals (SRM8_3), responsible sourcing (SRM8_4), transparency (SRM8_5), and compliance with regulations and standards (SRM8_6)).

## Digital twin architecture

The numbers number of criteria and sub-criteria concerned with the sustainability risk assessment and management in the supply chain is needed to reduce the static assessment and one-way assessment and to achieve the effectiveness assessment.

**Figure 1:**
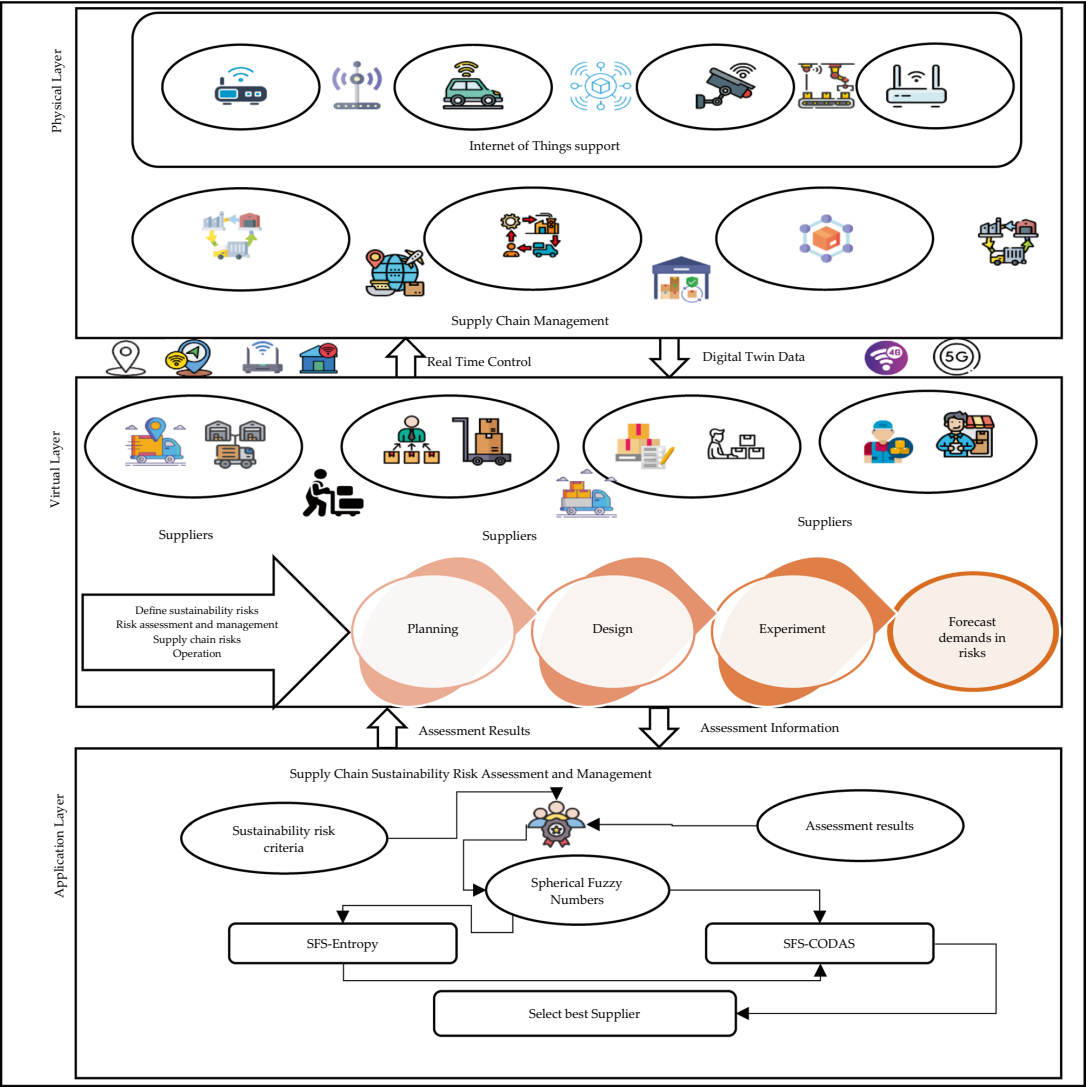
The architecture of digital twin.

We used the digital twin control system which is an extension of the digital twin mapping system to compute all factors of sustainable assessment. We construct a digital twin architecture for supply chain sustainability risk assessment and management and selection best supplier under risk assessment with the complete life cycle as shown in Fig. 1.

There are two methods used to supply the information and data for supply chain sustainability risk assessment and management intelligent assessment method, dynamic evolution, and technology of virtual mapping. The digital twin architecture of this study has three layers as physical layer, the virtual layer, and the application layer.

### Physical layer

The physical layer is a suitable key for data collection and real-world interaction. In the physical layer, the real-world physical evaluation and process in the SC include the transport vehicles, lines of production, and datasets. It is integrated with various IoT elements such as sensors, intelligent systems, and data gathering. These elements are used to compute the real-time data to help in assessment. The physical layer interacts with various instances, such as production lines.

### Virtual layer

This layer is used to create a virtual representation of the data that come from the physical layer such as physical assets and processes. The virtual layer is used to forecast the outcome of data based on previous data such as forecast the supply demand. This layer can be used for instance testing and planning.

### Application layer

This layer is used to assess the supply chain sustainability risk assessment and management based on a framework of the MCDM method. In the MCDM framework, the SFS is used to deal with various uncertainty data in the assessment process. The MCDM framework consists of two MCDM methods SFS-Entropy and SFS-CODAS methods. The SFS-Entropy method is used to compute the weights of criteria of supply chain sustainability risk assessment and management. The SFS-CODAS method is used to rank the suppliers and select the best one.

## Materials and methods

This section discusses the Steps of the proposed methodology for selecting the best supplier with supply chain sustainability risk assessment and management (SCSRAM). This section used the two MCDM methods under SFS: Entropy and CODAS. The SFS-Entropy method is used to compute the SCSRAM criteria weight, and the SFS-CODAS method is used to rank the suppliers and select the best one. The SFS was used in this study to deal with the uncertain data in the assessment process.

### SFS-Entropy-CODAS

**Figure 2:**
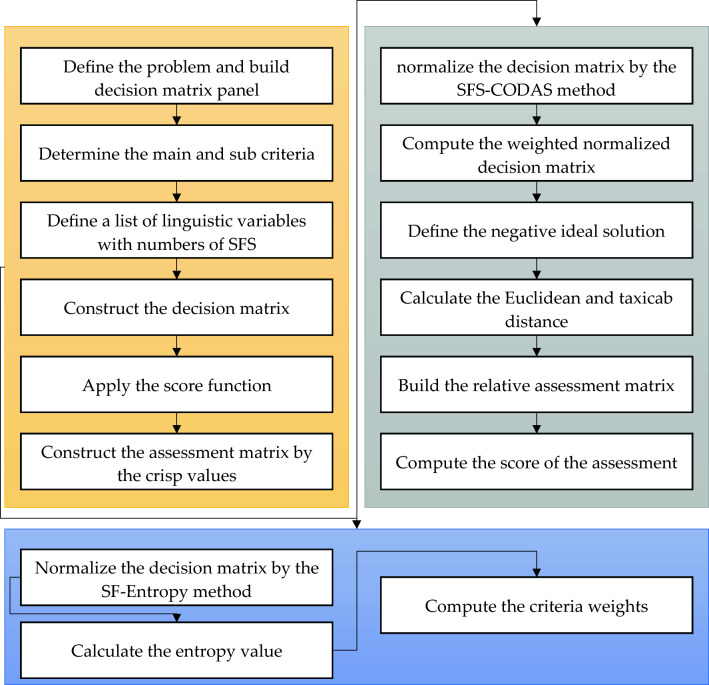
The SFS-Entropy-CODAS Framework.
- *Step 2* Determine the main and sub-criteria
  The primary and sub-criteria are determined in sustainability risks assessment and management through the study of the literature review and relevant studies. The symbols of criteria used in this study are $C_{j} = \left( {SRM_{1} ,SRM_{2} , \ldots SRM_{n} } \right), with \left( {j = 1,2 \ldots .n} \right)$, sub-criteria are $Sub_{j} = \left( {SRM_{11} ,SRM_{12} , \ldots SRM_{1n} } \right)$, and alternatives are $A_{i} = \left( {SRS_{1} ,SRS_{2} , \ldots SRS_{m} } \right), with \left( {i = 1,2 \ldots .m} \right)$. We define the criteria weight as a $W_{j} = \left( {w_{1} ,w_{1} , \ldots w_{n} } \right);where\, w_{j} > 0\, and \mathop \sum \limits_{j = 1}^{n} w_{j} = 1$.
- *Step 3* Define a list of linguistic variables with numbers of SFS
  We define a list of linguistic variables with SFS numbers (SFNs), as shown in Table 1. The experts use these variables and decision makers to evaluate the criteria and sub-criteria to compute the main and sub-criteria weight and evaluate the alternatives to select the best supplier in the SCSRAM.

- *Step 1* Define the problem
  The problem of this study is defined with a high accuracy with all details. The main criteria of the problem are defined, and all sub-criteria are defined in this step. Various rules are used to select the experts and decision-makers to evaluate the requirements and alternatives in this study. This study invited a committee of experts in Egypt gas firms to evaluate the criteria and alternatives based on their opinions. This committee includes twenty experts who have expertise in supply chain risk assessment and management. The experts have expertise between 15 and 25 years and have Ph.D. and M.Sc. academic degrees. The evaluating criteria, sub-criteria, and alternatives are evaluated based on the opinions of the experts and decision-makers. The data were chosen with high accuracy and effectiveness due to the large number of criteria and sub-criteria, and they need more time to be evaluated. Figure 2 shows the steps of the proposed method.

**Table 1:** The linguistic variables of SFS.

Linguistic variables	SFNs
Absolutely High	(0.9, 0.1, 0.1)
Very High	(0.8, 0.2, 0.2)
High	(0.7, 0.3, 0.3)
Moderate High	(0.6, 0.4, 0.4)
Equal	(0.5, 0.5, 0.5)
Moderate Low	(0.4, 0.6, 0.4)
Low	(0.3, 0.7, 0.3)
Very Low	(0.2, 0.8, 0.2)
Absolutely Low	(0.1, 0.9, 0.1)
- *Step 4* Construct the decision matrix
  The decision matrix is defined by experts' opinions on criteria, sub-criteria, and alternatives. The experts used the linguistic variables in Table 1 to build the decision matrix.
  1 $$ A = \left[ {\begin{array}{*{20}c} {\left( {x_{11} ,y_{11} , z_{11} } \right)} & \cdots & {\left( {x_{1n} ,y_{1n} , z_{1n} } \right)} \\ \vdots & \ddots & \vdots \\ {\left( {x_{m1} ,y_{m1} , z_{m1} } \right)} & \cdots & {\left( {x_{mn} ,y_{mn} , z_{mn} } \right)} \\ \end{array} } \right] $$
- *Step 5* Apply the score function
  We convert the SFNs into a crisp value by applying the score function, then the decision matrix is built based on the crisp values.
  2 $$ S\left( A \right) = x \times \left( {1 - y} \right) \times \left( {1 - z} \right) $$
  3 $$ A = \left[ {\begin{array}{*{20}c} {a_{11} } & \cdots & {a_{1n} } \\ \vdots & \ddots & \vdots \\ {a_{m1} } & \cdots & {a_{mn} } \\ \end{array} } \right] $$
- *Step 6* Apply the SFS-Entropy method, Normalize the decision matrix
  Shannon and Weaver suggested the entropy method used to compute the criteria weight. It is used to estimate the spreading values in decision-making. The entropy method is used in the probability theory and computes vague information or entropy. This method determines the objective weight for the main and sub-criteria.
  4 $$ N_{ij} = \frac{{a_{ij} }}{{\mathop \sum \nolimits_{i = 1}^{n} a_{ij} }} $$
- *Step 7* Calculate the entropy value
  The entropy value can be used to compute decision information in the normalized decision matrix and queued in every factor.
  5 $$ e_{j} = - \frac{1}{\log n }\mathop \sum \limits_{i = 1}^{n} N_{ij} \log \left( {N_{ij} } \right) $$
- *Step 8* Compute the criteria weights
  The objective weights of the criteria are computed
  6 $$ W_{j} = \frac{{d_{j} }}{{\mathop \sum \nolimits_{j = 1}^{m} d_{j} }} $$
  7 $$ d_{j} = 1 - e_{j} $$
- *Step 9* Apply the steps of the SFS-CODAS and normalize the decision matrix by the SFS-CODAS method
  Based on the crisp value in the decision matrix, we compute the normalized decision matrix by the SFS-CODAS method. The normalization matrix is computed for both benefit and cost criteria.
  8 $$ u_{ij} = \frac{{a_{ij} }}{{\mathop {\max }\limits_{i} a_{ij} }} benefit\;criteria $$
  9 $$ u_{ij} = \frac{{\mathop {\min }\limits_{i} a_{ij} }}{{a_{ij} }} cost\;criteria $$
- *Step 10* Compute the weighted normalized decision matrix.
  10 $$ Q_{ij} = W_{j} \times u_{ij} $$
- *Step 11* Define the negative ideal solution
  The negative ideal solution is defined for all criteria and sub-criteria.
  11 $$ NS = \mathop {\min }\limits_{i} q_{ij} $$
- *Step 12* Calculate the Euclidean and taxicab distance
  The Euclidean and taxicab distance are computed from NS and alternatives:
  12 $$ E_{i} = \sqrt {\mathop \sum \limits_{j = 1}^{n} \left( {q_{ij} - NS_{j} } \right)^{2} } $$
  13 $$ T_{i} = \mathop \sum \limits_{j = 1}^{n} \left| {q_{ij} - NS_{j} } \right|^{2} $$
- *Step 13* Build the relative assessment matrix
  14 $$ L_{is} = \left( {E_{i} - E_{s} } \right) + \left( {\beta \left( {E_{i} - E_{s} } \right) \times \left( {T_{i} - T_{s} } \right)} \right) $$
  where $s \in \left\{ {1,2, \ldots m} \right\}\;{\text{and}}\;\beta$ refers to the threshold function to define the equality of the alternatives by the distances.
- *Step 14* Compute the score of the assessment
  The assessment score is computed for all options. The options are ranked in descending order of $H_{i}$.
  15 $$ H_{i} = \mathop \sum \limits_{i = 1}^{n} L_{is} $$

## Application

This section applies the steps of the proposed method of the SFS-Entropy-CODAS method. This section discusses the results of the proposed method. This study proposed a framework for the selection of the best supplier of a gas company in Egypt. This section needs to choose the best supplier from ten suppliers. We used in this study 8 main criteria and 38 sub-criteria. The gas firm in Egypt has been established for more than 40 years. It has more than 500 employees. It provides gas services for local and international countries in the Middle East. This company tends to use digital technology in its operations to reduce risks in the SC, so the digital twin is integrated with SC to assess and manage risks and select the best supplier to achieve the firm's goals.

**Figure 3:**
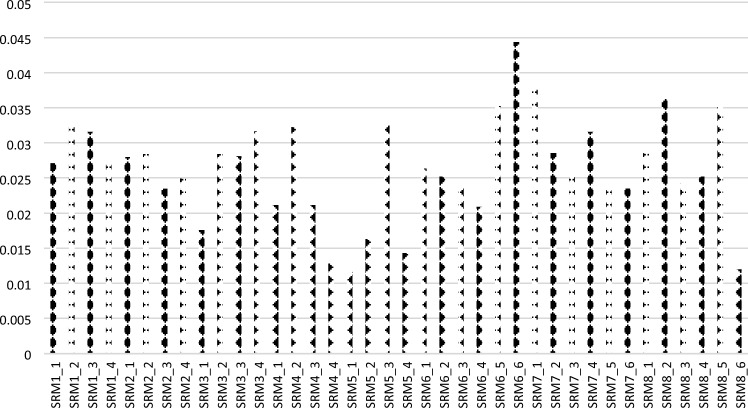
The criteria weights of supply chain suitability risk assessment and management.
- *Step 9* Apply the steps of the SFS-CODAS and normalize the decision matrix by the SFS-CODAS method by Eq. (8). All criteria are benefit criteria as shown in Table 2.

- *Step 1* Define the problem
  The details of the problem are defined and determined. The goal of this study, rank the suppliers to gas companies in Egypt based on supply chain sustainability risk assessment and management. The excerpts are selected based on their expertise in this field.
- *Step 2* Determine the main and sub-criteria
  8 main criteria and 38 sub-criteria are determined in this study. The main criteria are named supply risks (SRM_1_) (supply chain complexity (SRM_1_1_), single-source dependencies(SRM_1_2_), transportation vulnerabilities (SRM_1_3_), and contingency planning (SRM_1_4_)), financial risk (SRM_2_) (financial health (SRM_2_1_), creditworthiness (SRM_2_2_), bankruptcy risk (SRM_2_3_), and payment terms (SRM_2_4_)), political risk (SRM_3_) (geopolitical stability (SRM_3_1_), political unrest (SRM_3_2_), natural disasters (SRM_3_3_), and supply chain vulnerabilities in specific regions or countries (SRM_3_4_)), regulatory risk (SRM_4_) (regulatory requirements (SRM_4_1_), industry standards (SRM_4_2_), product safety (SRM_4_3_), and quality standards (SRM_4_4_)), technology risk (SRM_5_) (technology obsolescence (SRM_5_1_), cybersecurity vulnerabilities (SRM_5_2_), data privacy (SRM_5_3_), and intellectual property protection (SRM_5_4_)), environmental risk (SRM_6_) (greenhouse gas emissions (SRM_6_1_), water usage (SRM_6_2_), waste generation (SRM_6_3_), pollution (SRM_6_4_), deforestation (SRM_6_5_), and resource depletion (SRM_6_6_)), social risk (SRM_7_) (working conditions (SRM_7_1_), labor rights (SRM_7_2_), health and safety standards (SRM_7_3_), diversity (SRM_7_4_), equality (SRM_7_5_), and human rights (SRM_7_6_)), and ethical risk (SRM_8_) (corruption (SRM_8_1_), bribery (SRM_8_2_), conflict minerals (SRM_8_3_), responsible sourcing (SRM_8_4_), transparency (SRM_8_5_), and compliance with regulations and standards (SRM_8_6_)).
- *Step 3* Define a list of linguistic variables with numbers of SFS
  There are nine linguistic variables are used in this paper. For instance, (0.9,0.1,0.1) refers to the high. These variables are used by experts and decision-makers to evaluate the criteria, sub-criteria, and alternatives to define the weights of the criteria. The alternatives are SRS_1_, SRS_2_, SRS_3_, SRS_4_, SRS_5_, SRS_6_, SRS_7_, SRS_8_, SRS_9_, SRS_10_. Twenty experts evaluate the criteria and alternatives based on their opinions; then, they are gathered in a decision matrix to compute the criteria weights and rank the other options. The experts used the linguistic variables to evaluate the requirements and alternatives. Then, we replace these variables with their SFNs. Digital Twin SC can collect data from the physical layer by using IoT components and sensors. Experts then used these data to evaluate the criteria and alternatives. Big data analyses are then used to analyze these data. The data in the virtual layer can enable decision-makers and experts to make decisions by using digital twin factors. Experts used digital twins to provide a visualization representation of physical layers.
- *Step 4* Construct the decision matrix
  The decision matrix is built by Eq. (1) between criteria and alternatives by the opinions of experts and decision-makers. These elements are evaluated by the linguistic variables. The experts used the SFNs.
- *Step 5* Apply the score function
  The decision matrix values are converted to crisp value by applying the score function by Eq. (2). Then build the decision matrix by the crisp value by Eq. (3). For example, we let an Absolutely High, (0.9, 0.1, 0.1) be an SFN; then we applied the score function to compute the crisp value as 0.9*(1–0.1)*(1–0.1), then the crisp value is 0.7.
- *Step 6* Apply the SFS-Entropy method, Normalize the decision matrix
  The decision matrix is normalized by Eq. (4). The values of the decision matrix between criteria and alternatives are normalized. For example, we divided each value into crisp values by the sum of each column. The sum of the first column in the decision matrix is 3.7, then we divided the 0.729 by the 3.7, and then the result of the normalization is 0.193.
- *Step 7* Calculate the entropy value
  We compute the entropy valued by Eq. (5). For example, if the number of criteria is 38, then we compute the log (38) and multiply by the summation of the number of criteria and log (number of criteria).
- *Step 8* Compute the criteria weights
  The criteria weights are computed by Eqs. (6 and 7). Figure 3 shows the criteria weights. We show the supply risk has the highest importance followed by financial risk and the ethical risk has the lowest risk. In the global weight, we show that SRM_6_6_ and SRM_7_1_ have the highest weight and SRM_8_6_ has the lowest weight.

**Table 2:** The normalization decision matrix by the SFS-CODAS method.

	SRS1	SRS2	SRS3	SRS4	SRS5	SRS6	SRS7	SRS8	SRS9	SRS10
SRM1_1	1.000	0.044	0.132	0.702	1.000	0.702	0.296	0.171	0.132	1.000
SRM1_2	0.132	1.000	0.171	0.296	1.000	0.132	0.044	0.132	0.702	1.000
SRM1_3	0.702	0.044	0.132	0.132	1.000	0.171	0.296	0.132	0.702	1.000
SRM1_4	0.132	1.000	0.171	0.296	0.702	1.000	0.044	0.132	0.702	1.000
SRM2_1	0.132	1.000	0.171	0.296	0.702	0.296	0.044	0.132	0.702	1.000
SRM2_2	0.132	1.000	0.132	1.000	0.171	0.296	0.296	0.171	0.132	0.702
SRM2_3	0.132	1.000	0.171	0.296	0.171	0.296	0.132	0.702	1.000	0.702
SRM2_4	0.132	1.000	0.171	0.296	0.702	0.296	0.132	1.000	0.171	0.296
SRM3_1	1.000	0.422	0.244	0.188	0.422	0.244	1.000	0.422	0.244	0.188
SRM3_2	0.132	1.000	0.171	0.296	0.132	0.702	0.132	1.000	0.171	0.296
SRM3_3	0.702	0.702	0.296	0.171	0.132	0.171	0.044	0.132	0.702	1.000
SRM3_4	0.132	1.000	0.171	0.132	1.000	0.171	0.296	0.296	0.171	0.132
SRM4_1	0.702	0.296	0.171	0.132	0.702	0.296	0.171	0.132	0.702	1.000
SRM4_2	0.702	0.296	0.171	0.132	0.044	0.132	0.132	1.000	0.171	0.296
SRM4_3	0.702	0.296	0.171	0.132	0.702	0.296	0.171	0.132	1.000	0.702
SRM4_4	0.702	0.471	0.702	1.000	0.702	0.296	0.132	1.000	0.171	0.702
SRM5_1	0.702	0.471	0.702	1.000	0.702	0.702	0.296	0.171	0.132	0.702
SRM5_2	1.000	0.422	0.244	0.188	0.244	0.188	0.422	0.244	0.188	0.244
SRM5_3	0.044	0.132	0.702	1.000	0.132	0.702	0.296	0.171	0.132	0.171
SRM5_4	0.132	1.000	0.171	0.296	0.702	1.000	0.471	0.702	1.000	0.471
SRM6_1	0.471	0.044	0.132	0.702	1.000	0.702	0.132	1.000	0.171	0.296
SRM6_2	0.132	1.000	0.171	0.296	0.702	1.000	0.132	1.000	0.171	0.296
SRM6_3	0.132	1.000	0.171	0.132	1.000	0.171	0.296	0.471	0.702	1.000
SRM6_4	0.471	0.702	1.000	0.702	0.296	0.171	0.044	0.132	0.702	1.000
SRM6_5	0.044	0.132	0.702	1.000	0.702	0.044	0.132	1.000	0.171	0.296
SRM6_6	0.044	0.132	0.044	0.132	0.702	1.000	0.044	0.132	0.702	1.000
SRM7_1	0.044	0.044	0.132	0.702	1.000	0.471	0.044	0.132	0.702	1.000
SRM7_2	0.044	0.132	0.702	1.000	0.702	1.000	0.044	0.132	0.702	1.000
SRM7_3	0.044	0.132	0.702	1.000	0.702	1.000	0.702	0.296	0.171	0.132
SRM7_4	0.044	0.132	0.702	0.132	1.000	0.171	0.296	0.132	0.702	1.000
SRM7_5	0.132	1.000	0.171	0.296	0.702	1.000	0.702	0.296	0.171	0.132
SRM7_6	0.471	0.702	0.132	1.000	0.171	0.296	0.132	1.000	0.171	0.296
SRM8_1	0.471	0.702	1.000	0.044	0.132	0.702	0.044	0.132	0.702	1.000
SRM8_2	0.044	0.132	0.702	1.000	0.132	0.296	0.044	0.132	0.702	1.000
SRM8_3	0.471	0.702	0.044	0.132	0.702	1.000	0.702	0.296	0.171	0.132
SRM8_4	0.132	1.000	0.171	0.296	0.702	1.000	0.132	1.000	0.171	0.296
SRM8_5	0.044	0.132	0.702	1.000	1.000	0.044	0.132	0.702	1.000	0.132
SRM8_6	0.471	0.702	0.471	0.702	0.296	0.171	0.132	0.471	0.702	1.000
- *Step 10* Compute the weighted normalized decision matrix by Eqs. (10) by multiplying the weights of global criteria by the normalization decision matrix as shown in Table 3.

**Table 3:** The normalization decision matrix by the SFS-CODAS method.

	SRS1	SRS2	SRS3	SRS4	SRS5	SRS6	SRS7	SRS8	SRS9	SRS10
SRM1_1	0.027	0.001	0.004	0.019	0.027	0.019	0.008	0.005	0.004	0.027
SRM1_2	0.004	0.033	0.006	0.010	0.033	0.004	0.001	0.004	0.023	0.033
SRM1_3	0.022	0.001	0.004	0.004	0.032	0.005	0.009	0.004	0.022	0.032
SRM1_4	0.004	0.027	0.005	0.008	0.019	0.027	0.001	0.004	0.019	0.027
SRM2_1	0.004	0.028	0.005	0.008	0.020	0.008	0.001	0.004	0.020	0.028
SRM2_2	0.004	0.028	0.004	0.028	0.005	0.008	0.008	0.005	0.004	0.020
SRM2_3	0.003	0.023	0.004	0.007	0.004	0.007	0.003	0.016	0.023	0.016
SRM2_4	0.003	0.025	0.004	0.007	0.018	0.007	0.003	0.025	0.004	0.007
SRM3_1	0.018	0.007	0.004	0.003	0.007	0.004	0.018	0.007	0.004	0.003
SRM3_2	0.004	0.028	0.005	0.008	0.004	0.020	0.004	0.028	0.005	0.008
SRM3_3	0.020	0.020	0.008	0.005	0.004	0.005	0.001	0.004	0.020	0.028
SRM3_4	0.004	0.032	0.005	0.004	0.032	0.005	0.009	0.009	0.005	0.004
SRM4_1	0.015	0.006	0.004	0.003	0.015	0.006	0.004	0.003	0.015	0.021
SRM4_2	0.023	0.010	0.006	0.004	0.001	0.004	0.004	0.032	0.006	0.010
SRM4_3	0.015	0.006	0.004	0.003	0.015	0.006	0.004	0.003	0.021	0.015
SRM4_4	0.009	0.006	0.009	0.013	0.009	0.004	0.002	0.013	0.002	0.009
SRM5_1	0.008	0.005	0.008	0.012	0.008	0.008	0.003	0.002	0.002	0.008
SRM5_2	0.016	0.007	0.004	0.003	0.004	0.003	0.007	0.004	0.003	0.004
SRM5_3	0.001	0.004	0.023	0.033	0.004	0.023	0.010	0.006	0.004	0.006
SRM5_4	0.002	0.014	0.002	0.004	0.010	0.014	0.007	0.010	0.014	0.007
SRM6_1	0.012	0.001	0.003	0.018	0.026	0.018	0.003	0.026	0.005	0.008
SRM6_2	0.003	0.025	0.004	0.007	0.018	0.025	0.003	0.025	0.004	0.007
SRM6_3	0.003	0.023	0.004	0.003	0.023	0.004	0.007	0.011	0.016	0.023
SRM6_4	0.010	0.015	0.021	0.015	0.006	0.004	0.001	0.003	0.015	0.021
SRM6_5	0.002	0.005	0.025	0.035	0.025	0.002	0.005	0.035	0.006	0.010
SRM6_6	0.002	0.006	0.002	0.006	0.031	0.044	0.002	0.006	0.031	0.044
SRM7_1	0.002	0.002	0.005	0.027	0.038	0.018	0.002	0.005	0.027	0.038
SRM7_2	0.001	0.004	0.020	0.029	0.020	0.029	0.001	0.004	0.020	0.029
SRM7_3	0.001	0.003	0.018	0.026	0.018	0.026	0.018	0.008	0.004	0.003
SRM7_4	0.001	0.004	0.022	0.004	0.032	0.005	0.009	0.004	0.022	0.032
SRM7_5	0.003	0.023	0.004	0.007	0.016	0.023	0.016	0.007	0.004	0.003
SRM7_6	0.011	0.017	0.003	0.024	0.004	0.007	0.003	0.024	0.004	0.007
SRM8_1	0.013	0.020	0.029	0.001	0.004	0.020	0.001	0.004	0.020	0.029
SRM8_2	0.002	0.005	0.026	0.036	0.005	0.011	0.002	0.005	0.026	0.036
SRM8_3	0.011	0.017	0.001	0.003	0.017	0.024	0.017	0.007	0.004	0.003
SRM8_4	0.003	0.025	0.004	0.007	0.018	0.025	0.003	0.025	0.004	0.007
SRM8_5	0.002	0.005	0.025	0.035	0.035	0.002	0.005	0.025	0.035	0.005
SRM8_6	0.006	0.008	0.006	0.008	0.004	0.002	0.002	0.006	0.008	0.012
- *Step 11* Define the negative ideal solution for all criteria by Eq. (11)
- *Step 12* Calculate the Euclidean and taxicab distance for each alternative between NS values.
- *Step 13* Build the relative assessment matrix by Eq. (14). We set the $\beta$ value to 0.5.
- *Step 14* Compute the score of assessment by Eq. (15) as shown in Fig. 4. We show that supplier 10 is the best and supplier 7 is the worst.

**Figure 4:**
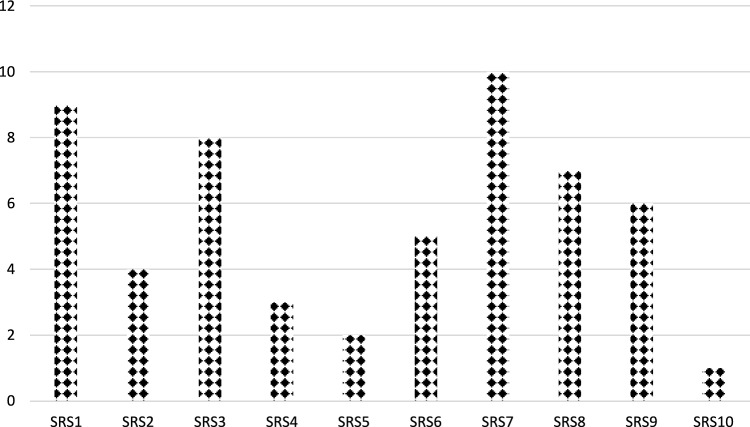
The assessment value for each alternative.

### Discussion

We discuss the results obtained from the proposed SFS-Entropy-CODAS method. This study's results are divided into two parts. In the first part, we compute the weights of supply chain sustainability risk assessment and management criteria for this study's main and sub-criteria. In the second part, we compute the rank of suppliers using the SFS-CODAS method.

We applied this study to select the best supplier for gas firm in Egypt. The committee of experts and decision-makers for this firm have expertise in the digital twin, decision-making, and supply chain, and they have academic degrees. These experts and decision-makers gathered the suitable criteria for this study. Then, they used the linguistic variables, as shown in Table 1, to evaluate the criteria and alternatives to their opinions. Then, we replaced these variables with spherical fuzzy numbers. Then, we applied the score function to convert the spherical fuzzy numbers into a crisp value. Then, we use the Entropy and CODAS methods to compute the criteria weights and rank the alternatives.

The weights of eight main criteria and 38 sub-criteria in this study are computed by the SFS-Entropy method. In the main criteria, we show that supply risk has a weight equal to 0.175 and is the most important, followed by financial risk, which has a weight equal to 0.154, environmental risk, which has a weight equal to 0.1243, and technology risk, which has a weight equal to 0.1240. The ethical risk, which has a weight equal to 0.0965, has the lowest weight.

In the supply risk sub-criteria, we show that SRM1_2 is the highest weight, equal to 0.03322, followed by SRM1_3, equal to 0.0315, SRM1_1, equal to 0.027092, and SRM1_4, equal to 0.02709.

In the financial risk sub-criteria, we show that SRM2_1 has the highest weight, 0.0279, followed by SRM2_2, 0.028, SRM2_4, 0.024, and SRM2_3, 0.023.

In the political risk sub-criteria, we show that SRM3_4 has the highest weight, equal to 0.0316, followed by SRM3_2, which has a weight of 0.0284, SRM3_3, which has a weight of 0.08086, and SRM3_1, which has a weight of 0.017585.

In the regulatory risk sub-criteria, we show that SRM4_2 is the highest weight which has a weight equal to 0.032237, followed by SRM4_1, which has a weight equal to 0.021163, and SRM4_3, which has a weight equal to 0.021163, and the SRM4_4 is the lowest weight which has a weight equal to 0.012798.

In the technology risk sub-criteria, we show that SRM5_3 is the highest weight, with a weight equal to 0.032544, followed by SRM5_2, which has a weight equal to 0.016302, and SRM5_4, which has a weight equal to 0.014308. SRM5_1 is the lowest weight, with a weight equal to 0.011627.

In the environmental risk sub-criteria, we show that the SRM6_6 is the highest weight, equal to 0.044391, followed by the SRM6_5, equal to 0.03524, and the SRM6_1, equal to 0.026319. The SRM6_4 is the lowest weight, equal to 0.02091.

In the social risk sub-criteria, we show that SRM7_1 has the highest weight, equal to 0.038275, followed by SRM7_4, which has a weight equal to 0.0315, SRM7_2, which has a weight equal to 0.028558, and SRM7_5, which has a weight equal to 0.023491.

In the ethical risk sub-criteria, we show that the SRM8_2 has the highest weight, 0.036441, followed by the SRM8_5, 0.035086, and SRM8_1, 0.028514. The SRM8_6 has the lowest weight, 0.01199.

In the global weights of criteria, we show that SRM6_6 has the highest weight, equal to 0.044391, followed by SRM7_1, which has a weight equal to 0.038275, SRM8_2, which has a weight equal to 0.036441, and SRM6_5, which has a weight equal to 0.036441. The SRM5_1 has the lowest weight, equal to 0.011627.

In the ranking of alternatives, we used ten suppliers to be ranked by the SFS-CODAS method. We set the $\beta$ value with 0.5. Then we compute the assessment score. We show that Supplier 10 is the best followed by Supplier 5 and Supplier 7.

## Analysis

This section dividing into two parts. In the first part, we show the sensitivity analysis of this work. In the second part, we show the comparative analysis with other MCDM methods.

### Sensitivity analysis

The sensitivity analysis shows the stability and consistency of the obtained results. Some problems are considered when ranking the alternatives based on the opinions of experts and decision-makers based on criteria weights. In this study, the sensitivity analysis is divided into two parts. In the first part, we change the parameter's value in the CODAS method. In the second part, we change the weights of the criteria.

**Figure 5:**
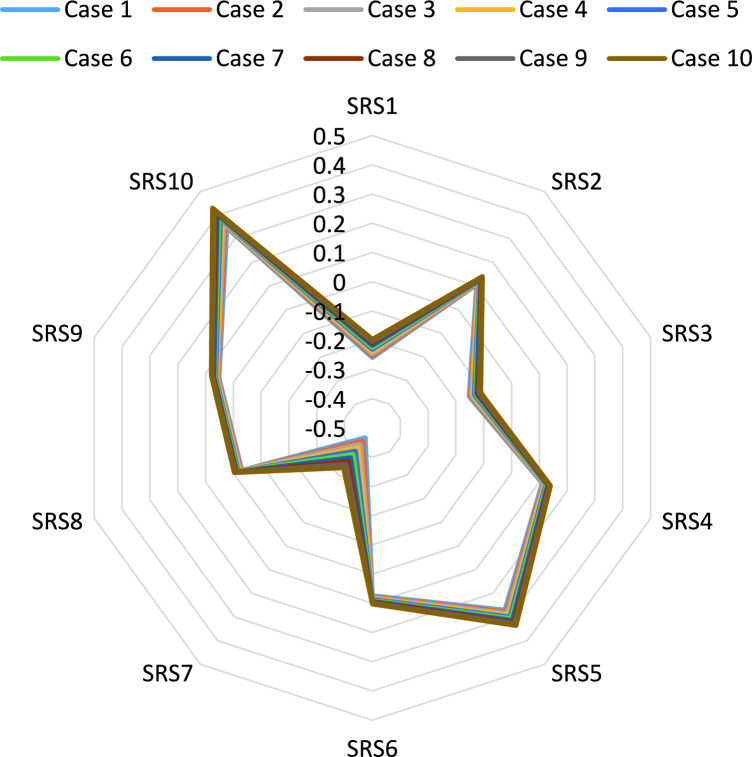
The rank of suppliers under ten cases of $\beta$ parameter.

In the first part, we change the parameter $\beta$ value between 0.1 and 1 and rank the alternatives. In the first case, we put the value $\beta$ 0.1; in the second case, we put the parameter $\beta$ 0.2, and so on. The supplier's rank is based on ten cases, as shown in Fig. 5. The results show all ranks in all cases are the same. Supplier ten is the best, and supplier seven is the worst.

**Figure 6:**
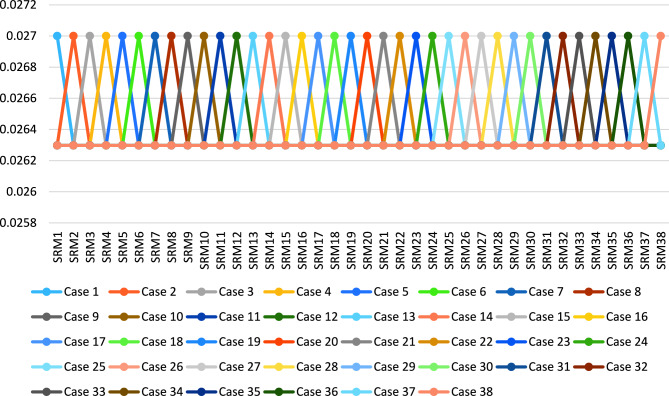
The 38 cases in changing of criteria weights.

In the second part, we change the criteria weights by 38 cases. First, we put one criterion with 0.027 weights, and all other criteria weights equal, as shown in Fig. 6.

**Figure 7:**
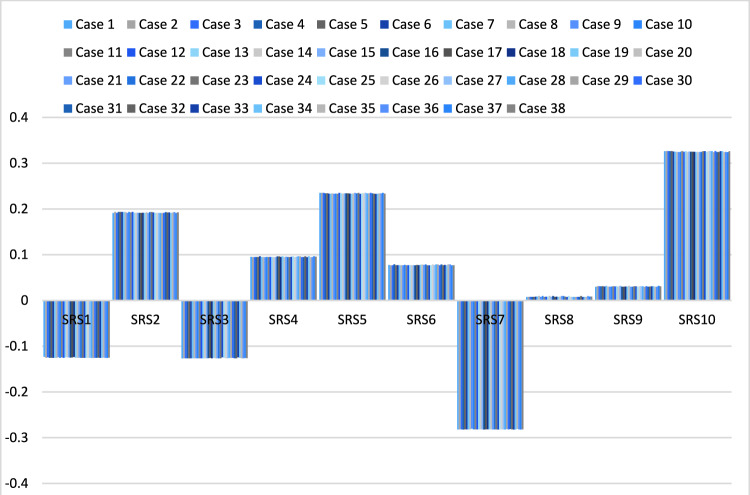
The rank of alternatives under 38 cases of criteria weights.

Then, we rank the ten suppliers under 38 cases in weight. We show all ranks are the same. Supplier ten has the highest rank, and supplier seven has the lowest, as shown in Fig. 7. We conclude that the results from the SFS-Entropy-CODAS method are stable and consistent under different changes.

### Comparative analysis

**Figure 8:**
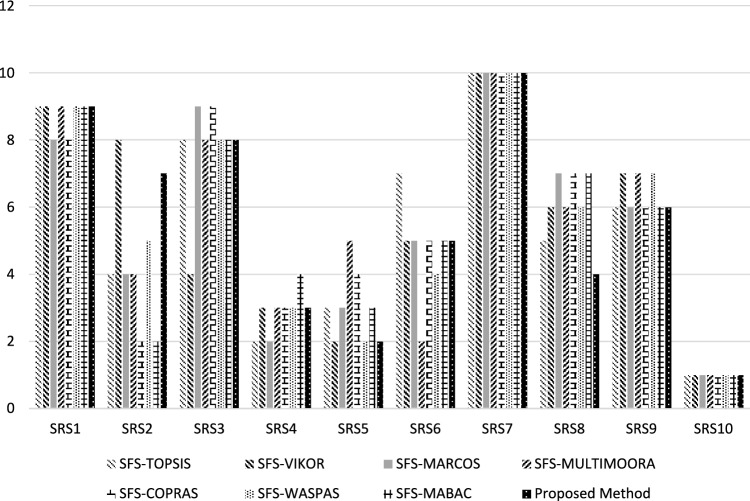
The rank of suppliers under comparative analysis.

This part compares the proposed SFS-Entropy-CODAS method with other MCDM methods under SFS to show its effectiveness and validity. The proposed method is compared with SFS-TOPSIS^61^, SFS-MABAC^62^, SFS-MARCOS^63^, SFS-WASPAS^64^, SFS-VIKOR^65^, SFS-MULTIMOORA^66^, and SFS-COPRAS^67^ methods to rank the ten suppliers under supply chain sustainability risk assessment and management. Figure 8 shows the rank of suppliers under different MCDM methods. All MCDM methods agreed that supplier 10 is the best and supplier 7 is the worst. We compute the correlation between the proposed methodology and other comparative MCDM methods, in which the correlation value is greater than 0.6. This indicates there is a high correlation. We show the correlation coefficient between the proposed methodology and the SFS-TOPSIS method is 0.90, between the proposed methodology and SFS-VIKOR is 0.86, between the proposed methodology and SFS-MARCOS is 0.86, between the proposed method and SFS-MULTIMOORA is 0.80, between proposed methodology and SFS-COPRAS is 0.75, between proposed methodology and SFS-WASPAS is 0.93, between proposed methodology and SFS-MABAC is 0.78.

## Challenges

The supply chain sustainability risk assessment and management challenges must be presented and discussed in Egypt gas firm. These challenges have come from various factors, such as the responsibilities of social and ecological aims, the complexity of future supply chain goals, and global market goals. We can organize these challenges in supply chain sustainability risk assessment and management as follows:

- The supply chain in Egypt gas firm has various customers and stakeholders, and these stakeholders must achieve their goals and targets. This makes the supply chain in Egypt gas firm complex due to multiple goals, and all goals should be achieved.
- There are various complex factors and criteria when dealing with risk assessment and management with sustainability due to ecological impacts, supply risks, and social risks.
- Integrating sustainability in risk assessment and management in the supply chain in Egypt gas firm can require more operational costs; hence, this operational cost needs to be balanced with various costs, such as investment costs in a competitive market. So, this leads to new risk factors in assessment.
- The sustainability factors in risk assessment and management need high technologies such as IoT devices and digital twin usage.

## Managerial implications

The managerial implications in supply chain suitability risk assessment and management are suitable for managers, decision-makers, and governance in Egypt gas firm. The managerial implication includes:

1. Egypt gas firm managers need to obtain the sustainability target in the organization with supply chain strategies to compute sustainability aims.
2. Risk assessment and identification are important for Egypt gas firm managers to demonstrate potential impacts on the supply chain.
3. Egypt gas firm used digital twin technologies, such as IoT elements and digital twins, to enhance supply chain visibility. These technologies can achieve real-time data.
4. Identifying the supply chain risks in Egypt's gas firm achieved sustainable performance and built trust among its stakeholders.
5. Digital twins can enhance the supply chain particles in Egypt gas firms over time by monitoring performance with the sustainability target and risk management.

## Conclusions

The assessment of risks in the supply chain is a critical task in business management, as well as increasing awareness of the environment, social and economic factors, and supply chain operations. There are various risks related to the supply chain, such as supply, financial, environmental, and others. Identifying potential risks can help managers and organizations. An integrated MCDM methodology is used under a spherical fuzzy set. This methodology has two methods: SFS-Entropy and SFS-CODAS. The SFS-Entropy was used to compute the weights of eight main criteria and 38 sub-criteria related to supply chain sustainability risk assessment and management. Then, the SFS-CODAS method was used to rank and select the best supplier from the ten suppliers used in this study. Digital SC can reduce risks, enhance SC operations, and increase production. Digital SC is related to IoT devices, sensors, and big data analytics tools. These tools are used to reduce SC risks. The results of this study show that supply risks have the highest importance, followed by financial risks and environmental risks, and the lowest important is ethical risk. The results show that supplier ten is the best and supplier seven is the worst. The sensitivity analysis proposed 38 cases in the criteria weights change to show a stability rank of alternatives. The sensitivity analysis is employed in this study to show the stability of the results. The comparative analysis shows the suggested methodology is robust compared with various MCDM methods.

The proposed methodology can be applied in various applications of supply chains, such as food supply chains, to manage food and reduce food wastage. Various MCDM methods, such as AHP and DEMATEL methods, can be used in this study to obtain the relationships between criteria and compute the criteria weights.

### Supplementary Information

Supplementary Information.

## Data Availability

The datasets used and/or analysed during the current study available from the corresponding author on reasonable request.

## Abbreviations

SFNs: Spherical Fuzzy Numbers
MCDM: Multi-Criteria Decision-Making
CODAS: COmbinative Distance-based Assessment
TOPSIS: Technique for Order Preference by Similarity to Ideal Solution
VIKOR: VIšeKriterijumska Optimizacija I Kompromisno Resenje
WASPAS: Weighted Aggregated Sum Product Assessment
MARCOS: Measurement Alternatives and Ranking according to COmpromise Solution
COPRAS: Complex Proportional Assessment
MULTIMOORA: Fully Multiplicative Form
