# Supplementary material for: Digital twin and fuzzy framework for supply chain sustainability risk assessment and management in supplier selection
Source: Sci Rep. 2024 Jul 31;14:17718. doi: 10.1038/s41598-024-67226-z (PMC11291748; doi:10.1038/s41598-024-67226-z)
Supplement: Supplementary file 1 — Supplementary Information. [file 41598_2024_67226_MOESM1_ESM.docx]

Decision matrix

|  | SRS1 | SRS2 | SRS3 | SRS4 | SRS5 | SRS6 | SRS7 | SRS8 | SRS9 | SRS10 |
| --- | --- | --- | --- | --- | --- | --- | --- | --- | --- | --- |
| SRM1_1 | (0.9, 0.1, 0.1) | (0.1, 0.9, 0.1) | (0.4, 0.6, 0.4) | (0.8, 0.2, 0.2) | (0.9, 0.1, 0.1) | (0.8, 0.2, 0.2) | (0.6, 0.4, 0.4) | (0.5, 0.5, 0.5) | (0.4, 0.6, 0.4) | (0.9, 0.1, 0.1) |
| SRM1_2 | (0.4, 0.6, 0.4) | (0.9, 0.1, 0.1) | (0.5, 0.5, 0.5) | (0.6, 0.4, 0.4) | (0.9, 0.1, 0.1) | (0.4, 0.6, 0.4) | (0.1, 0.9, 0.1) | (0.4, 0.6, 0.4) | (0.8, 0.2, 0.2) | (0.9, 0.1, 0.1) |
| SRM1_3 | (0.8, 0.2, 0.2) | (0.1, 0.9, 0.1) | (0.4, 0.6, 0.4) | (0.4, 0.6, 0.4) | (0.9, 0.1, 0.1) | (0.5, 0.5, 0.5) | (0.6, 0.4, 0.4) | (0.4, 0.6, 0.4) | (0.8, 0.2, 0.2) | (0.9, 0.1, 0.1) |
| SRM1_4 | (0.4, 0.6, 0.4) | (0.9, 0.1, 0.1) | (0.5, 0.5, 0.5) | (0.6, 0.4, 0.4) | (0.8, 0.2, 0.2) | (0.9, 0.1, 0.1) | (0.1, 0.9, 0.1) | (0.4, 0.6, 0.4) | (0.8, 0.2, 0.2) | (0.9, 0.1, 0.1) |
| SRM2_1 | (0.4, 0.6, 0.4) | (0.9, 0.1, 0.1) | (0.5, 0.5, 0.5) | (0.6, 0.4, 0.4) | (0.8, 0.2, 0.2) | (0.6, 0.4, 0.4) | (0.1, 0.9, 0.1) | (0.4, 0.6, 0.4) | (0.8, 0.2, 0.2) | (0.9, 0.1, 0.1) |
| SRM2_2 | (0.4, 0.6, 0.4) | (0.9, 0.1, 0.1) | (0.4, 0.6, 0.4) | (0.9, 0.1, 0.1) | (0.5, 0.5, 0.5) | (0.6, 0.4, 0.4) | (0.6, 0.4, 0.4) | (0.5, 0.5, 0.5) | (0.4, 0.6, 0.4) | (0.8, 0.2, 0.2) |
| SRM2_3 | (0.4, 0.6, 0.4) | (0.9, 0.1, 0.1) | (0.5, 0.5, 0.5) | (0.6, 0.4, 0.4) | (0.5, 0.5, 0.5) | (0.6, 0.4, 0.4) | (0.4, 0.6, 0.4) | (0.8, 0.2, 0.2) | (0.9, 0.1, 0.1) | (0.8, 0.2, 0.2) |
| SRM2_4 | (0.4, 0.6, 0.4) | (0.9, 0.1, 0.1) | (0.5, 0.5, 0.5) | (0.6, 0.4, 0.4) | (0.8, 0.2, 0.2) | (0.6, 0.4, 0.4) | (0.4, 0.6, 0.4) | (0.9, 0.1, 0.1) | (0.5, 0.5, 0.5) | (0.6, 0.4, 0.4) |
| SRM3_1 | (0.8, 0.2, 0.2) | (0.6, 0.4, 0.4) | (0.5, 0.5, 0.5) | (0.4, 0.6, 0.4) | (0.6, 0.4, 0.4) | (0.5, 0.5, 0.5) | (0.8, 0.2, 0.2) | (0.6, 0.4, 0.4) | (0.5, 0.5, 0.5) | (0.4, 0.6, 0.4) |
| SRM3_2 | (0.4, 0.6, 0.4) | (0.9, 0.1, 0.1) | (0.5, 0.5, 0.5) | (0.6, 0.4, 0.4) | (0.4, 0.6, 0.4) | (0.8, 0.2, 0.2) | (0.4, 0.6, 0.4) | (0.9, 0.1, 0.1) | (0.5, 0.5, 0.5) | (0.6, 0.4, 0.4) |
| SRM3_3 | (0.8, 0.2, 0.2) | (0.8, 0.2, 0.2) | (0.6, 0.4, 0.4) | (0.5, 0.5, 0.5) | (0.4, 0.6, 0.4) | (0.5, 0.5, 0.5) | (0.1, 0.9, 0.1) | (0.4, 0.6, 0.4) | (0.8, 0.2, 0.2) | (0.9, 0.1, 0.1) |
| SRM3_4 | (0.4, 0.6, 0.4) | (0.9, 0.1, 0.1) | (0.5, 0.5, 0.5) | (0.4, 0.6, 0.4) | (0.9, 0.1, 0.1) | (0.5, 0.5, 0.5) | (0.6, 0.4, 0.4) | (0.6, 0.4, 0.4) | (0.5, 0.5, 0.5) | (0.4, 0.6, 0.4) |
| SRM4_1 | (0.8, 0.2, 0.2) | (0.6, 0.4, 0.4) | (0.5, 0.5, 0.5) | (0.4, 0.6, 0.4) | (0.8, 0.2, 0.2) | (0.6, 0.4, 0.4) | (0.5, 0.5, 0.5) | (0.4, 0.6, 0.4) | (0.8, 0.2, 0.2) | (0.9, 0.1, 0.1) |
| SRM4_2 | (0.8, 0.2, 0.2) | (0.6, 0.4, 0.4) | (0.5, 0.5, 0.5) | (0.4, 0.6, 0.4) | (0.1, 0.9, 0.1) | (0.4, 0.6, 0.4) | (0.4, 0.6, 0.4) | (0.9, 0.1, 0.1) | (0.5, 0.5, 0.5) | (0.6, 0.4, 0.4) |
| SRM4_3 | (0.8, 0.2, 0.2) | (0.6, 0.4, 0.4) | (0.5, 0.5, 0.5) | (0.4, 0.6, 0.4) | (0.8, 0.2, 0.2) | (0.6, 0.4, 0.4) | (0.5, 0.5, 0.5) | (0.4, 0.6, 0.4) | (0.9, 0.1, 0.1) | (0.8, 0.2, 0.2) |
| SRM4_4 | (0.8, 0.2, 0.2) | (0.7, 0.3, 0.3) | (0.8, 0.2, 0.2) | (0.9, 0.1, 0.1) | (0.8, 0.2, 0.2) | (0.6, 0.4, 0.4) | (0.4, 0.6, 0.4) | (0.9, 0.1, 0.1) | (0.5, 0.5, 0.5) | (0.8, 0.2, 0.2) |
| SRM5_1 | (0.8, 0.2, 0.2) | (0.7, 0.3, 0.3) | (0.8, 0.2, 0.2) | (0.9, 0.1, 0.1) | (0.8, 0.2, 0.2) | (0.8, 0.2, 0.2) | (0.6, 0.4, 0.4) | (0.5, 0.5, 0.5) | (0.4, 0.6, 0.4) | (0.8, 0.2, 0.2) |
| SRM5_2 | (0.8, 0.2, 0.2) | (0.6, 0.4, 0.4) | (0.5, 0.5, 0.5) | (0.4, 0.6, 0.4) | (0.5, 0.5, 0.5) | (0.4, 0.6, 0.4) | (0.6, 0.4, 0.4) | (0.5, 0.5, 0.5) | (0.4, 0.6, 0.4) | (0.5, 0.5, 0.5) |
| SRM5_3 | (0.1, 0.9, 0.1) | (0.4, 0.6, 0.4) | (0.8, 0.2, 0.2) | (0.9, 0.1, 0.1) | (0.4, 0.6, 0.4) | (0.8, 0.2, 0.2) | (0.6, 0.4, 0.4) | (0.5, 0.5, 0.5) | (0.4, 0.6, 0.4) | (0.5, 0.5, 0.5) |
| SRM5_4 | (0.4, 0.6, 0.4) | (0.9, 0.1, 0.1) | (0.5, 0.5, 0.5) | (0.6, 0.4, 0.4) | (0.8, 0.2, 0.2) | (0.9, 0.1, 0.1) | (0.7, 0.3, 0.3) | (0.8, 0.2, 0.2) | (0.9, 0.1, 0.1) | (0.7, 0.3, 0.3) |
| SRM6_1 | (0.7, 0.3, 0.3) | (0.1, 0.9, 0.1) | (0.4, 0.6, 0.4) | (0.8, 0.2, 0.2) | (0.9, 0.1, 0.1) | (0.8, 0.2, 0.2) | (0.4, 0.6, 0.4) | (0.9, 0.1, 0.1) | (0.5, 0.5, 0.5) | (0.6, 0.4, 0.4) |
| SRM6_2 | (0.4, 0.6, 0.4) | (0.9, 0.1, 0.1) | (0.5, 0.5, 0.5) | (0.6, 0.4, 0.4) | (0.8, 0.2, 0.2) | (0.9, 0.1, 0.1) | (0.4, 0.6, 0.4) | (0.9, 0.1, 0.1) | (0.5, 0.5, 0.5) | (0.6, 0.4, 0.4) |
| SRM6_3 | (0.4, 0.6, 0.4) | (0.9, 0.1, 0.1) | (0.5, 0.5, 0.5) | (0.4, 0.6, 0.4) | (0.9, 0.1, 0.1) | (0.5, 0.5, 0.5) | (0.6, 0.4, 0.4) | (0.7, 0.3, 0.3) | (0.8, 0.2, 0.2) | (0.9, 0.1, 0.1) |
| SRM6_4 | (0.7, 0.3, 0.3) | (0.8, 0.2, 0.2) | (0.9, 0.1, 0.1) | (0.8, 0.2, 0.2) | (0.6, 0.4, 0.4) | (0.5, 0.5, 0.5) | (0.1, 0.9, 0.1) | (0.4, 0.6, 0.4) | (0.8, 0.2, 0.2) | (0.9, 0.1, 0.1) |
| SRM6_5 | (0.1, 0.9, 0.1) | (0.4, 0.6, 0.4) | (0.8, 0.2, 0.2) | (0.9, 0.1, 0.1) | (0.8, 0.2, 0.2) | (0.1, 0.9, 0.1) | (0.4, 0.6, 0.4) | (0.9, 0.1, 0.1) | (0.5, 0.5, 0.5) | (0.6, 0.4, 0.4) |
| SRM6_6 | (0.1, 0.9, 0.1) | (0.4, 0.6, 0.4) | (0.1, 0.9, 0.1) | (0.4, 0.6, 0.4) | (0.8, 0.2, 0.2) | (0.9, 0.1, 0.1) | (0.1, 0.9, 0.1) | (0.4, 0.6, 0.4) | (0.8, 0.2, 0.2) | (0.9, 0.1, 0.1) |
| SRM7_1 | (0.1, 0.9, 0.1) | (0.1, 0.9, 0.1) | (0.4, 0.6, 0.4) | (0.8, 0.2, 0.2) | (0.9, 0.1, 0.1) | (0.7, 0.3, 0.3) | (0.1, 0.9, 0.1) | (0.4, 0.6, 0.4) | (0.8, 0.2, 0.2) | (0.9, 0.1, 0.1) |
| SRM7_2 | (0.1, 0.9, 0.1) | (0.4, 0.6, 0.4) | (0.8, 0.2, 0.2) | (0.9, 0.1, 0.1) | (0.8, 0.2, 0.2) | (0.9, 0.1, 0.1) | (0.1, 0.9, 0.1) | (0.4, 0.6, 0.4) | (0.8, 0.2, 0.2) | (0.9, 0.1, 0.1) |
| SRM7_3 | (0.1, 0.9, 0.1) | (0.4, 0.6, 0.4) | (0.8, 0.2, 0.2) | (0.9, 0.1, 0.1) | (0.8, 0.2, 0.2) | (0.9, 0.1, 0.1) | (0.8, 0.2, 0.2) | (0.6, 0.4, 0.4) | (0.5, 0.5, 0.5) | (0.4, 0.6, 0.4) |
| SRM7_4 | (0.1, 0.9, 0.1) | (0.4, 0.6, 0.4) | (0.8, 0.2, 0.2) | (0.4, 0.6, 0.4) | (0.9, 0.1, 0.1) | (0.5, 0.5, 0.5) | (0.6, 0.4, 0.4) | (0.4, 0.6, 0.4) | (0.8, 0.2, 0.2) | (0.9, 0.1, 0.1) |
| SRM7_5 | (0.4, 0.6, 0.4) | (0.9, 0.1, 0.1) | (0.5, 0.5, 0.5) | (0.6, 0.4, 0.4) | (0.8, 0.2, 0.2) | (0.9, 0.1, 0.1) | (0.8, 0.2, 0.2) | (0.6, 0.4, 0.4) | (0.5, 0.5, 0.5) | (0.4, 0.6, 0.4) |
| SRM7_6 | (0.7, 0.3, 0.3) | (0.8, 0.2, 0.2) | (0.4, 0.6, 0.4) | (0.9, 0.1, 0.1) | (0.5, 0.5, 0.5) | (0.6, 0.4, 0.4) | (0.4, 0.6, 0.4) | (0.9, 0.1, 0.1) | (0.5, 0.5, 0.5) | (0.6, 0.4, 0.4) |
| SRM8_1 | (0.7, 0.3, 0.3) | (0.8, 0.2, 0.2) | (0.9, 0.1, 0.1) | (0.1, 0.9, 0.1) | (0.4, 0.6, 0.4) | (0.8, 0.2, 0.2) | (0.1, 0.9, 0.1) | (0.4, 0.6, 0.4) | (0.8, 0.2, 0.2) | (0.9, 0.1, 0.1) |
| SRM8_2 | (0.1, 0.9, 0.1) | (0.4, 0.6, 0.4) | (0.8, 0.2, 0.2) | (0.9, 0.1, 0.1) | (0.4, 0.6, 0.4) | (0.6, 0.4, 0.4) | (0.1, 0.9, 0.1) | (0.4, 0.6, 0.4) | (0.8, 0.2, 0.2) | (0.9, 0.1, 0.1) |
| SRM8_3 | (0.7, 0.3, 0.3) | (0.8, 0.2, 0.2) | (0.1, 0.9, 0.1) | (0.4, 0.6, 0.4) | (0.8, 0.2, 0.2) | (0.9, 0.1, 0.1) | (0.8, 0.2, 0.2) | (0.6, 0.4, 0.4) | (0.5, 0.5, 0.5) | (0.4, 0.6, 0.4) |
| SRM8_4 | (0.4, 0.6, 0.4) | (0.9, 0.1, 0.1) | (0.5, 0.5, 0.5) | (0.6, 0.4, 0.4) | (0.8, 0.2, 0.2) | (0.9, 0.1, 0.1) | (0.4, 0.6, 0.4) | (0.9, 0.1, 0.1) | (0.5, 0.5, 0.5) | (0.6, 0.4, 0.4) |
| SRM8_5 | (0.1, 0.9, 0.1) | (0.4, 0.6, 0.4) | (0.8, 0.2, 0.2) | (0.9, 0.1, 0.1) | (0.9, 0.1, 0.1) | (0.1, 0.9, 0.1) | (0.4, 0.6, 0.4) | (0.8, 0.2, 0.2) | (0.9, 0.1, 0.1) | (0.4, 0.6, 0.4) |
| SRM8_6 | (0.7, 0.3, 0.3) | (0.8, 0.2, 0.2) | (0.7, 0.3, 0.3) | (0.8, 0.2, 0.2) | (0.6, 0.4, 0.4) | (0.5, 0.5, 0.5) | (0.4, 0.6, 0.4) | (0.7, 0.3, 0.3) | (0.8, 0.2, 0.2) | (0.9, 0.1, 0.1) |

Normalize decision matrix by Entropy Method

|  | SRS1 | SRS2 | SRS3 | SRS4 | SRS5 | SRS6 | SRS7 | SRS8 | SRS9 | SRS10 |
| --- | --- | --- | --- | --- | --- | --- | --- | --- | --- | --- |
| SRM1_1 | 0.193061 | 0.008475 | 0.025424 | 0.135593 | 0.193061 | 0.135593 | 0.057203 | 0.033104 | 0.025424 | 0.193061 |
| SRM1_2 | 0.028571 | 0.216964 | 0.037202 | 0.064286 | 0.216964 | 0.028571 | 0.009524 | 0.028571 | 0.152381 | 0.216964 |
| SRM1_3 | 0.162902 | 0.010181 | 0.030544 | 0.030544 | 0.231944 | 0.039771 | 0.068724 | 0.030544 | 0.162902 | 0.231944 |
| SRM1_4 | 0.025424 | 0.193061 | 0.033104 | 0.057203 | 0.135593 | 0.193061 | 0.008475 | 0.025424 | 0.135593 | 0.193061 |
| SRM2_1 | 0.029421 | 0.223414 | 0.038308 | 0.066197 | 0.156911 | 0.066197 | 0.009807 | 0.029421 | 0.156911 | 0.223414 |
| SRM2_2 | 0.032653 | 0.247959 | 0.032653 | 0.247959 | 0.042517 | 0.073469 | 0.073469 | 0.042517 | 0.032653 | 0.17415 |
| SRM2_3 | 0.028605 | 0.217223 | 0.037247 | 0.064362 | 0.037247 | 0.064362 | 0.028605 | 0.152563 | 0.217223 | 0.152563 |
| SRM2_4 | 0.031373 | 0.238235 | 0.04085 | 0.070588 | 0.16732 | 0.070588 | 0.031373 | 0.238235 | 0.04085 | 0.070588 |
| SRM3_1 | 0.228674 | 0.096472 | 0.055828 | 0.042876 | 0.096472 | 0.055828 | 0.228674 | 0.096472 | 0.055828 | 0.042876 |
| SRM3_2 | 0.032653 | 0.247959 | 0.042517 | 0.073469 | 0.032653 | 0.17415 | 0.032653 | 0.247959 | 0.042517 | 0.073469 |
| SRM3_3 | 0.173266 | 0.173266 | 0.073096 | 0.042301 | 0.032487 | 0.042301 | 0.010829 | 0.032487 | 0.173266 | 0.246701 |
| SRM3_4 | 0.037603 | 0.285546 | 0.048962 | 0.037603 | 0.285546 | 0.048962 | 0.084606 | 0.084606 | 0.048962 | 0.037603 |
| SRM4_1 | 0.163109 | 0.068812 | 0.039822 | 0.030583 | 0.163109 | 0.068812 | 0.039822 | 0.030583 | 0.163109 | 0.23224 |
| SRM4_2 | 0.228266 | 0.0963 | 0.055729 | 0.0428 | 0.014267 | 0.0428 | 0.0428 | 0.325011 | 0.055729 | 0.0963 |
| SRM4_3 | 0.163109 | 0.068812 | 0.039822 | 0.030583 | 0.163109 | 0.068812 | 0.039822 | 0.030583 | 0.23224 | 0.163109 |
| SRM4_4 | 0.119459 | 0.080028 | 0.119459 | 0.170089 | 0.119459 | 0.050397 | 0.022399 | 0.170089 | 0.029165 | 0.119459 |
| SRM5_1 | 0.125829 | 0.084296 | 0.125829 | 0.179159 | 0.125829 | 0.125829 | 0.053084 | 0.03072 | 0.023593 | 0.125829 |
| SRM5_2 | 0.295612 | 0.124711 | 0.072171 | 0.055427 | 0.072171 | 0.055427 | 0.124711 | 0.072171 | 0.055427 | 0.072171 |
| SRM5_3 | 0.012603 | 0.03781 | 0.201654 | 0.287121 | 0.03781 | 0.201654 | 0.085073 | 0.049232 | 0.03781 | 0.049232 |
| SRM5_4 | 0.02215 | 0.168205 | 0.028842 | 0.049838 | 0.118136 | 0.168205 | 0.079142 | 0.118136 | 0.168205 | 0.079142 |
| SRM6_1 | 0.10118 | 0.00944 | 0.028319 | 0.151032 | 0.215044 | 0.151032 | 0.028319 | 0.215044 | 0.036873 | 0.063717 |
| SRM6_2 | 0.026868 | 0.20403 | 0.034985 | 0.060453 | 0.143297 | 0.20403 | 0.026868 | 0.20403 | 0.034985 | 0.060453 |
| SRM6_3 | 0.025946 | 0.197027 | 0.033784 | 0.025946 | 0.197027 | 0.033784 | 0.058378 | 0.092703 | 0.138378 | 0.197027 |
| SRM6_4 | 0.090121 | 0.134524 | 0.19154 | 0.134524 | 0.056752 | 0.032843 | 0.008408 | 0.025223 | 0.134524 | 0.19154 |
| SRM6_5 | 0.010393 | 0.031179 | 0.166288 | 0.236765 | 0.166288 | 0.010393 | 0.031179 | 0.236765 | 0.040598 | 0.070153 |
| SRM6_6 | 0.011165 | 0.033496 | 0.011165 | 0.033496 | 0.178646 | 0.254361 | 0.011165 | 0.033496 | 0.178646 | 0.254361 |
| SRM7_1 | 0.010279 | 0.010279 | 0.030838 | 0.164472 | 0.234179 | 0.110183 | 0.010279 | 0.030838 | 0.164472 | 0.234179 |
| SRM7_2 | 0.008042 | 0.024127 | 0.128676 | 0.183212 | 0.128676 | 0.183212 | 0.008042 | 0.024127 | 0.128676 | 0.183212 |
| SRM7_3 | 0.008991 | 0.026974 | 0.143861 | 0.204833 | 0.143861 | 0.204833 | 0.143861 | 0.060691 | 0.035122 | 0.026974 |
| SRM7_4 | 0.010181 | 0.030544 | 0.162902 | 0.030544 | 0.231944 | 0.039771 | 0.068724 | 0.030544 | 0.162902 | 0.231944 |
| SRM7_5 | 0.028605 | 0.217223 | 0.037247 | 0.064362 | 0.152563 | 0.217223 | 0.152563 | 0.064362 | 0.037247 | 0.028605 |
| SRM7_6 | 0.107625 | 0.160653 | 0.030122 | 0.228742 | 0.039222 | 0.067775 | 0.030122 | 0.228742 | 0.039222 | 0.067775 |
| SRM8_1 | 0.095463 | 0.142499 | 0.202895 | 0.008906 | 0.026719 | 0.142499 | 0.008906 | 0.026719 | 0.142499 | 0.202895 |
| SRM8_2 | 0.010492 | 0.031475 | 0.167869 | 0.239016 | 0.031475 | 0.07082 | 0.010492 | 0.031475 | 0.167869 | 0.239016 |
| SRM8_3 | 0.1081 | 0.161361 | 0.010085 | 0.030255 | 0.161361 | 0.229751 | 0.161361 | 0.068074 | 0.039395 | 0.030255 |
| SRM8_4 | 0.026868 | 0.20403 | 0.034985 | 0.060453 | 0.143297 | 0.20403 | 0.026868 | 0.20403 | 0.034985 | 0.060453 |
| SRM8_5 | 0.008981 | 0.026944 | 0.143699 | 0.204603 | 0.204603 | 0.008981 | 0.026944 | 0.143699 | 0.204603 | 0.026944 |
| SRM8_6 | 0.091932 | 0.137229 | 0.091932 | 0.137229 | 0.057893 | 0.033503 | 0.02573 | 0.091932 | 0.137229 | 0.19539 |

Normalize decision matrix by CODAS Method

|  | SRS1 | SRS2 | SRS3 | SRS4 | SRS5 | SRS6 | SRS7 | SRS8 | SRS9 | SRS10 |
| --- | --- | --- | --- | --- | --- | --- | --- | --- | --- | --- |
| SRM1_1 | 1 | 0.043896 | 0.131687 | 0.702332 | 1 | 0.702332 | 0.296296 | 0.171468 | 0.131687 | 1 |
| SRM1_2 | 0.131687 | 1 | 0.171468 | 0.296296 | 1 | 0.131687 | 0.043896 | 0.131687 | 0.702332 | 1 |
| SRM1_3 | 0.702332 | 0.043896 | 0.131687 | 0.131687 | 1 | 0.171468 | 0.296296 | 0.131687 | 0.702332 | 1 |
| SRM1_4 | 0.131687 | 1 | 0.171468 | 0.296296 | 0.702332 | 1 | 0.043896 | 0.131687 | 0.702332 | 1 |
| SRM2_1 | 0.131687 | 1 | 0.171468 | 0.296296 | 0.702332 | 0.296296 | 0.043896 | 0.131687 | 0.702332 | 1 |
| SRM2_2 | 0.131687 | 1 | 0.131687 | 1 | 0.171468 | 0.296296 | 0.296296 | 0.171468 | 0.131687 | 0.702332 |
| SRM2_3 | 0.131687 | 1 | 0.171468 | 0.296296 | 0.171468 | 0.296296 | 0.131687 | 0.702332 | 1 | 0.702332 |
| SRM2_4 | 0.131687 | 1 | 0.171468 | 0.296296 | 0.702332 | 0.296296 | 0.131687 | 1 | 0.171468 | 0.296296 |
| SRM3_1 | 1 | 0.421875 | 0.244141 | 0.1875 | 0.421875 | 0.244141 | 1 | 0.421875 | 0.244141 | 0.1875 |
| SRM3_2 | 0.131687 | 1 | 0.171468 | 0.296296 | 0.131687 | 0.702332 | 0.131687 | 1 | 0.171468 | 0.296296 |
| SRM3_3 | 0.702332 | 0.702332 | 0.296296 | 0.171468 | 0.131687 | 0.171468 | 0.043896 | 0.131687 | 0.702332 | 1 |
| SRM3_4 | 0.131687 | 1 | 0.171468 | 0.131687 | 1 | 0.171468 | 0.296296 | 0.296296 | 0.171468 | 0.131687 |
| SRM4_1 | 0.702332 | 0.296296 | 0.171468 | 0.131687 | 0.702332 | 0.296296 | 0.171468 | 0.131687 | 0.702332 | 1 |
| SRM4_2 | 0.702332 | 0.296296 | 0.171468 | 0.131687 | 0.043896 | 0.131687 | 0.131687 | 1 | 0.171468 | 0.296296 |
| SRM4_3 | 0.702332 | 0.296296 | 0.171468 | 0.131687 | 0.702332 | 0.296296 | 0.171468 | 0.131687 | 1 | 0.702332 |
| SRM4_4 | 0.702332 | 0.470508 | 0.702332 | 1 | 0.702332 | 0.296296 | 0.131687 | 1 | 0.171468 | 0.702332 |
| SRM5_1 | 0.702332 | 0.470508 | 0.702332 | 1 | 0.702332 | 0.702332 | 0.296296 | 0.171468 | 0.131687 | 0.702332 |
| SRM5_2 | 1 | 0.421875 | 0.244141 | 0.1875 | 0.244141 | 0.1875 | 0.421875 | 0.244141 | 0.1875 | 0.244141 |
| SRM5_3 | 0.043896 | 0.131687 | 0.702332 | 1 | 0.131687 | 0.702332 | 0.296296 | 0.171468 | 0.131687 | 0.171468 |
| SRM5_4 | 0.131687 | 1 | 0.171468 | 0.296296 | 0.702332 | 1 | 0.470508 | 0.702332 | 1 | 0.470508 |
| SRM6_1 | 0.470508 | 0.043896 | 0.131687 | 0.702332 | 1 | 0.702332 | 0.131687 | 1 | 0.171468 | 0.296296 |
| SRM6_2 | 0.131687 | 1 | 0.171468 | 0.296296 | 0.702332 | 1 | 0.131687 | 1 | 0.171468 | 0.296296 |
| SRM6_3 | 0.131687 | 1 | 0.171468 | 0.131687 | 1 | 0.171468 | 0.296296 | 0.470508 | 0.702332 | 1 |
| SRM6_4 | 0.470508 | 0.702332 | 1 | 0.702332 | 0.296296 | 0.171468 | 0.043896 | 0.131687 | 0.702332 | 1 |
| SRM6_5 | 0.043896 | 0.131687 | 0.702332 | 1 | 0.702332 | 0.043896 | 0.131687 | 1 | 0.171468 | 0.296296 |
| SRM6_6 | 0.043896 | 0.131687 | 0.043896 | 0.131687 | 0.702332 | 1 | 0.043896 | 0.131687 | 0.702332 | 1 |
| SRM7_1 | 0.043896 | 0.043896 | 0.131687 | 0.702332 | 1 | 0.470508 | 0.043896 | 0.131687 | 0.702332 | 1 |
| SRM7_2 | 0.043896 | 0.131687 | 0.702332 | 1 | 0.702332 | 1 | 0.043896 | 0.131687 | 0.702332 | 1 |
| SRM7_3 | 0.043896 | 0.131687 | 0.702332 | 1 | 0.702332 | 1 | 0.702332 | 0.296296 | 0.171468 | 0.131687 |
| SRM7_4 | 0.043896 | 0.131687 | 0.702332 | 0.131687 | 1 | 0.171468 | 0.296296 | 0.131687 | 0.702332 | 1 |
| SRM7_5 | 0.131687 | 1 | 0.171468 | 0.296296 | 0.702332 | 1 | 0.702332 | 0.296296 | 0.171468 | 0.131687 |
| SRM7_6 | 0.470508 | 0.702332 | 0.131687 | 1 | 0.171468 | 0.296296 | 0.131687 | 1 | 0.171468 | 0.296296 |
| SRM8_1 | 0.470508 | 0.702332 | 1 | 0.043896 | 0.131687 | 0.702332 | 0.043896 | 0.131687 | 0.702332 | 1 |
| SRM8_2 | 0.043896 | 0.131687 | 0.702332 | 1 | 0.131687 | 0.296296 | 0.043896 | 0.131687 | 0.702332 | 1 |
| SRM8_3 | 0.470508 | 0.702332 | 0.043896 | 0.131687 | 0.702332 | 1 | 0.702332 | 0.296296 | 0.171468 | 0.131687 |
| SRM8_4 | 0.131687 | 1 | 0.171468 | 0.296296 | 0.702332 | 1 | 0.131687 | 1 | 0.171468 | 0.296296 |
| SRM8_5 | 0.043896 | 0.131687 | 0.702332 | 1 | 1 | 0.043896 | 0.131687 | 0.702332 | 1 | 0.131687 |
| SRM8_6 | 0.470508 | 0.702332 | 0.470508 | 0.702332 | 0.296296 | 0.171468 | 0.131687 | 0.470508 | 0.702332 | 1 |

Weighted normalize decision matrix by CODAS Method

|  | SRS1 | SRS2 | SRS3 | SRS4 | SRS5 | SRS6 | SRS7 | SRS8 | SRS9 | SRS10 |
| --- | --- | --- | --- | --- | --- | --- | --- | --- | --- | --- |
| SRM1_1 | 0.026297 | 0.001154 | 0.003463 | 0.018469 | 0.026297 | 0.018469 | 0.007792 | 0.004509 | 0.003463 | 0.026297 |
| SRM1_2 | 0.003463 | 0.026297 | 0.004509 | 0.007792 | 0.026297 | 0.003463 | 0.001154 | 0.003463 | 0.018469 | 0.026297 |
| SRM1_3 | 0.018469 | 0.001154 | 0.003463 | 0.003463 | 0.026297 | 0.004509 | 0.007792 | 0.003463 | 0.018469 | 0.026297 |
| SRM1_4 | 0.003463 | 0.026297 | 0.004509 | 0.007792 | 0.018469 | 0.026297 | 0.001154 | 0.003463 | 0.018469 | 0.026297 |
| SRM2_1 | 0.003463 | 0.026297 | 0.004509 | 0.007792 | 0.018469 | 0.007792 | 0.001154 | 0.003463 | 0.018469 | 0.026297 |
| SRM2_2 | 0.003463 | 0.026297 | 0.003463 | 0.026297 | 0.004509 | 0.007792 | 0.007792 | 0.004509 | 0.003463 | 0.018469 |
| SRM2_3 | 0.003463 | 0.026297 | 0.004509 | 0.007792 | 0.004509 | 0.007792 | 0.003463 | 0.018469 | 0.026297 | 0.018469 |
| SRM2_4 | 0.003463 | 0.026297 | 0.004509 | 0.007792 | 0.018469 | 0.007792 | 0.003463 | 0.026297 | 0.004509 | 0.007792 |
| SRM3_1 | 0.026297 | 0.011094 | 0.00642 | 0.004931 | 0.011094 | 0.00642 | 0.026297 | 0.011094 | 0.00642 | 0.004931 |
| SRM3_2 | 0.003463 | 0.026297 | 0.004509 | 0.007792 | 0.003463 | 0.018469 | 0.003463 | 0.026297 | 0.004509 | 0.007792 |
| SRM3_3 | 0.018469 | 0.018469 | 0.007792 | 0.004509 | 0.003463 | 0.004509 | 0.001154 | 0.003463 | 0.018469 | 0.026297 |
| SRM3_4 | 0.003463 | 0.026297 | 0.004509 | 0.003463 | 0.026297 | 0.004509 | 0.007792 | 0.007792 | 0.004509 | 0.003463 |
| SRM4_1 | 0.018469 | 0.007792 | 0.004509 | 0.003463 | 0.018469 | 0.007792 | 0.004509 | 0.003463 | 0.018469 | 0.026297 |
| SRM4_2 | 0.018469 | 0.007792 | 0.004509 | 0.003463 | 0.001154 | 0.003463 | 0.003463 | 0.026297 | 0.004509 | 0.007792 |
| SRM4_3 | 0.018469 | 0.007792 | 0.004509 | 0.003463 | 0.018469 | 0.007792 | 0.004509 | 0.003463 | 0.026297 | 0.018469 |
| SRM4_4 | 0.018469 | 0.012373 | 0.018469 | 0.026297 | 0.018469 | 0.007792 | 0.003463 | 0.026297 | 0.004509 | 0.018469 |
| SRM5_1 | 0.018469 | 0.012373 | 0.018469 | 0.026297 | 0.018469 | 0.018469 | 0.007792 | 0.004509 | 0.003463 | 0.018469 |
| SRM5_2 | 0.026297 | 0.011094 | 0.00642 | 0.004931 | 0.00642 | 0.004931 | 0.011094 | 0.00642 | 0.004931 | 0.00642 |
| SRM5_3 | 0.001154 | 0.003463 | 0.018469 | 0.026297 | 0.003463 | 0.018469 | 0.007792 | 0.004509 | 0.003463 | 0.004509 |
| SRM5_4 | 0.003463 | 0.026297 | 0.004509 | 0.007792 | 0.018469 | 0.026297 | 0.012373 | 0.018469 | 0.026297 | 0.012373 |
| SRM6_1 | 0.012373 | 0.001154 | 0.003463 | 0.018469 | 0.026297 | 0.018469 | 0.003463 | 0.026297 | 0.004509 | 0.007792 |
| SRM6_2 | 0.003463 | 0.026297 | 0.004509 | 0.007792 | 0.018469 | 0.026297 | 0.003463 | 0.026297 | 0.004509 | 0.007792 |
| SRM6_3 | 0.003463 | 0.026297 | 0.004509 | 0.003463 | 0.026297 | 0.004509 | 0.007792 | 0.012373 | 0.018469 | 0.026297 |
| SRM6_4 | 0.012373 | 0.018469 | 0.026297 | 0.018469 | 0.007792 | 0.004509 | 0.001154 | 0.003463 | 0.018469 | 0.026297 |
| SRM6_5 | 0.001154 | 0.003463 | 0.018469 | 0.026297 | 0.018469 | 0.001154 | 0.003463 | 0.026297 | 0.004509 | 0.007792 |
| SRM6_6 | 0.001154 | 0.003463 | 0.001154 | 0.003463 | 0.018469 | 0.026297 | 0.001154 | 0.003463 | 0.018469 | 0.026297 |
| SRM7_1 | 0.001154 | 0.001154 | 0.003463 | 0.018469 | 0.026297 | 0.012373 | 0.001154 | 0.003463 | 0.018469 | 0.026297 |
| SRM7_2 | 0.001154 | 0.003463 | 0.018469 | 0.026297 | 0.018469 | 0.026297 | 0.001154 | 0.003463 | 0.018469 | 0.026297 |
| SRM7_3 | 0.001154 | 0.003463 | 0.018469 | 0.026297 | 0.018469 | 0.026297 | 0.018469 | 0.007792 | 0.004509 | 0.003463 |
| SRM7_4 | 0.001154 | 0.003463 | 0.018469 | 0.003463 | 0.026297 | 0.004509 | 0.007792 | 0.003463 | 0.018469 | 0.026297 |
| SRM7_5 | 0.003463 | 0.026297 | 0.004509 | 0.007792 | 0.018469 | 0.026297 | 0.018469 | 0.007792 | 0.004509 | 0.003463 |
| SRM7_6 | 0.012373 | 0.018469 | 0.003463 | 0.026297 | 0.004509 | 0.007792 | 0.003463 | 0.026297 | 0.004509 | 0.007792 |
| SRM8_1 | 0.012373 | 0.018469 | 0.026297 | 0.001154 | 0.003463 | 0.018469 | 0.001154 | 0.003463 | 0.018469 | 0.026297 |
| SRM8_2 | 0.001154 | 0.003463 | 0.018469 | 0.026297 | 0.003463 | 0.007792 | 0.001154 | 0.003463 | 0.018469 | 0.026297 |
| SRM8_3 | 0.012373 | 0.018469 | 0.001154 | 0.003463 | 0.018469 | 0.026297 | 0.018469 | 0.007792 | 0.004509 | 0.003463 |
| SRM8_4 | 0.003463 | 0.026297 | 0.004509 | 0.007792 | 0.018469 | 0.026297 | 0.003463 | 0.026297 | 0.004509 | 0.007792 |
| SRM8_5 | 0.001154 | 0.003463 | 0.018469 | 0.026297 | 0.026297 | 0.001154 | 0.003463 | 0.018469 | 0.026297 | 0.003463 |
| SRM8_6 | 0.012704 | 0.018963 | 0.012704 | 0.018963 | 0.008 | 0.00463 | 0.003556 | 0.012704 | 0.018963 | 0.027 |

Two distances by CODAS Method

|  | SRS1 | SRS2 | SRS3 | SRS4 | SRS5 | SRS6 | SRS7 | SRS8 | SRS9 | SRS10 |
| --- | --- | --- | --- | --- | --- | --- | --- | --- | --- | --- |
| SRM1_1 | 0.000632 | 0 | 5.33E-06 | 0.0003 | 0.000632 | 0.0003 | 4.41E-05 | 1.13E-05 | 5.33E-06 | 0.000632 |
| SRM1_2 | 5.33E-06 | 0.000632 | 1.13E-05 | 4.41E-05 | 0.000632 | 5.33E-06 | 0 | 5.33E-06 | 0.0003 | 0.000632 |
| SRM1_3 | 0.0003 | 0 | 5.33E-06 | 5.33E-06 | 0.000632 | 1.13E-05 | 4.41E-05 | 5.33E-06 | 0.0003 | 0.000632 |
| SRM1_4 | 5.33E-06 | 0.000632 | 1.13E-05 | 4.41E-05 | 0.0003 | 0.000632 | 0 | 5.33E-06 | 0.0003 | 0.000632 |
| SRM2_1 | 5.33E-06 | 0.000632 | 1.13E-05 | 4.41E-05 | 0.0003 | 4.41E-05 | 0 | 5.33E-06 | 0.0003 | 0.000632 |
| SRM2_2 | 0 | 0.000521 | 0 | 0.000521 | 1.09E-06 | 1.87E-05 | 1.87E-05 | 1.09E-06 | 0 | 0.000225 |
| SRM2_3 | 0 | 0.000521 | 1.09E-06 | 1.87E-05 | 1.09E-06 | 1.87E-05 | 0 | 0.000225 | 0.000521 | 0.000225 |
| SRM2_4 | 0 | 0.000521 | 1.09E-06 | 1.87E-05 | 0.000225 | 1.87E-05 | 0 | 0.000521 | 1.09E-06 | 1.87E-05 |
| SRM3_1 | 0.000457 | 3.8E-05 | 2.22E-06 | 0 | 3.8E-05 | 2.22E-06 | 0.000457 | 3.8E-05 | 2.22E-06 | 0 |
| SRM3_2 | 0 | 0.000521 | 1.09E-06 | 1.87E-05 | 0 | 0.000225 | 0 | 0.000521 | 1.09E-06 | 1.87E-05 |
| SRM3_3 | 0.0003 | 0.0003 | 4.41E-05 | 1.13E-05 | 5.33E-06 | 1.13E-05 | 0 | 5.33E-06 | 0.0003 | 0.000632 |
| SRM3_4 | 0 | 0.000521 | 1.09E-06 | 0 | 0.000521 | 1.09E-06 | 1.87E-05 | 1.87E-05 | 1.09E-06 | 0 |
| SRM4_1 | 0.000225 | 1.87E-05 | 1.09E-06 | 0 | 0.000225 | 1.87E-05 | 1.09E-06 | 0 | 0.000225 | 0.000521 |
| SRM4_2 | 0.0003 | 4.41E-05 | 1.13E-05 | 5.33E-06 | 0 | 5.33E-06 | 5.33E-06 | 0.000632 | 1.13E-05 | 4.41E-05 |
| SRM4_3 | 0.000225 | 1.87E-05 | 1.09E-06 | 0 | 0.000225 | 1.87E-05 | 1.09E-06 | 0 | 0.000521 | 0.000225 |
| SRM4_4 | 0.000225 | 7.94E-05 | 0.000225 | 0.000521 | 0.000225 | 1.87E-05 | 0 | 0.000521 | 1.09E-06 | 0.000225 |
| SRM5_1 | 0.000225 | 7.94E-05 | 0.000225 | 0.000521 | 0.000225 | 0.000225 | 1.87E-05 | 1.09E-06 | 0 | 0.000225 |
| SRM5_2 | 0.000457 | 3.8E-05 | 2.22E-06 | 0 | 2.22E-06 | 0 | 3.8E-05 | 2.22E-06 | 0 | 2.22E-06 |
| SRM5_3 | 0 | 5.33E-06 | 0.0003 | 0.000632 | 5.33E-06 | 0.0003 | 4.41E-05 | 1.13E-05 | 5.33E-06 | 1.13E-05 |
| SRM5_4 | 0 | 0.000521 | 1.09E-06 | 1.87E-05 | 0.000225 | 0.000521 | 7.94E-05 | 0.000225 | 0.000521 | 7.94E-05 |
| SRM6_1 | 0.000126 | 0 | 5.33E-06 | 0.0003 | 0.000632 | 0.0003 | 5.33E-06 | 0.000632 | 1.13E-05 | 4.41E-05 |
| SRM6_2 | 0 | 0.000521 | 1.09E-06 | 1.87E-05 | 0.000225 | 0.000521 | 0 | 0.000521 | 1.09E-06 | 1.87E-05 |
| SRM6_3 | 0 | 0.000521 | 1.09E-06 | 0 | 0.000521 | 1.09E-06 | 1.87E-05 | 7.94E-05 | 0.000225 | 0.000521 |
| SRM6_4 | 0.000126 | 0.0003 | 0.000632 | 0.0003 | 4.41E-05 | 1.13E-05 | 0 | 5.33E-06 | 0.0003 | 0.000632 |
| SRM6_5 | 0 | 5.33E-06 | 0.0003 | 0.000632 | 0.0003 | 0 | 5.33E-06 | 0.000632 | 1.13E-05 | 4.41E-05 |
| SRM6_6 | 0 | 5.33E-06 | 0 | 5.33E-06 | 0.0003 | 0.000632 | 0 | 5.33E-06 | 0.0003 | 0.000632 |
| SRM7_1 | 0 | 0 | 5.33E-06 | 0.0003 | 0.000632 | 0.000126 | 0 | 5.33E-06 | 0.0003 | 0.000632 |
| SRM7_2 | 0 | 5.33E-06 | 0.0003 | 0.000632 | 0.0003 | 0.000632 | 0 | 5.33E-06 | 0.0003 | 0.000632 |
| SRM7_3 | 0 | 5.33E-06 | 0.0003 | 0.000632 | 0.0003 | 0.000632 | 0.0003 | 4.41E-05 | 1.13E-05 | 5.33E-06 |
| SRM7_4 | 0 | 5.33E-06 | 0.0003 | 5.33E-06 | 0.000632 | 1.13E-05 | 4.41E-05 | 5.33E-06 | 0.0003 | 0.000632 |
| SRM7_5 | 0 | 0.000521 | 1.09E-06 | 1.87E-05 | 0.000225 | 0.000521 | 0.000225 | 1.87E-05 | 1.09E-06 | 0 |
| SRM7_6 | 7.94E-05 | 0.000225 | 0 | 0.000521 | 1.09E-06 | 1.87E-05 | 0 | 0.000521 | 1.09E-06 | 1.87E-05 |
| SRM8_1 | 0.000126 | 0.0003 | 0.000632 | 0 | 5.33E-06 | 0.0003 | 0 | 5.33E-06 | 0.0003 | 0.000632 |
| SRM8_2 | 0 | 5.33E-06 | 0.0003 | 0.000632 | 5.33E-06 | 4.41E-05 | 0 | 5.33E-06 | 0.0003 | 0.000632 |
| SRM8_3 | 0.000126 | 0.0003 | 0 | 5.33E-06 | 0.0003 | 0.000632 | 0.0003 | 4.41E-05 | 1.13E-05 | 5.33E-06 |
| SRM8_4 | 0 | 0.000521 | 1.09E-06 | 1.87E-05 | 0.000225 | 0.000521 | 0 | 0.000521 | 1.09E-06 | 1.87E-05 |
| SRM8_5 | 0 | 5.33E-06 | 0.0003 | 0.000632 | 0.000632 | 0 | 5.33E-06 | 0.0003 | 0.000632 | 5.33E-06 |
| SRM8_6 | 8.37E-05 | 0.000237 | 8.37E-05 | 0.000237 | 1.98E-05 | 1.15E-06 | 0 | 8.37E-05 | 0.000237 | 0.00055 |

Two distances by CODAS Method

|  | SRS1 | SRS2 | SRS3 | SRS4 | SRS5 | SRS6 | SRS7 | SRS8 | SRS9 | SRS10 |
| --- | --- | --- | --- | --- | --- | --- | --- | --- | --- | --- |
| SRM1_1 | 0.025143 | 0 | 0.002309 | 0.017315 | 0.025143 | 0.017315 | 0.006637 | 0.003355 | 0.002309 | 0.025143 |
| SRM1_2 | 0.002309 | 0.025143 | 0.003355 | 0.006637 | 0.025143 | 0.002309 | 0 | 0.002309 | 0.017315 | 0.025143 |
| SRM1_3 | 0.017315 | 0 | 0.002309 | 0.002309 | 0.025143 | 0.003355 | 0.006637 | 0.002309 | 0.017315 | 0.025143 |
| SRM1_4 | 0.002309 | 0.025143 | 0.003355 | 0.006637 | 0.017315 | 0.025143 | 0 | 0.002309 | 0.017315 | 0.025143 |
| SRM2_1 | 0.002309 | 0.025143 | 0.003355 | 0.006637 | 0.017315 | 0.006637 | 0 | 0.002309 | 0.017315 | 0.025143 |
| SRM2_2 | 0 | 0.022834 | 0 | 0.022834 | 0.001046 | 0.004329 | 0.004329 | 0.001046 | 0 | 0.015006 |
| SRM2_3 | 0 | 0.022834 | 0.001046 | 0.004329 | 0.001046 | 0.004329 | 0 | 0.015006 | 0.022834 | 0.015006 |
| SRM2_4 | 0 | 0.022834 | 0.001046 | 0.004329 | 0.015006 | 0.004329 | 0 | 0.022834 | 0.001046 | 0.004329 |
| SRM3_1 | 0.021367 | 0.006163 | 0.001489 | 0 | 0.006163 | 0.001489 | 0.021367 | 0.006163 | 0.001489 | 0 |
| SRM3_2 | 0 | 0.022834 | 0.001046 | 0.004329 | 0 | 0.015006 | 0 | 0.022834 | 0.001046 | 0.004329 |
| SRM3_3 | 0.017315 | 0.017315 | 0.006637 | 0.003355 | 0.002309 | 0.003355 | 0 | 0.002309 | 0.017315 | 0.025143 |
| SRM3_4 | 0 | 0.022834 | 0.001046 | 0 | 0.022834 | 0.001046 | 0.004329 | 0.004329 | 0.001046 | 0 |
| SRM4_1 | 0.015006 | 0.004329 | 0.001046 | 0 | 0.015006 | 0.004329 | 0.001046 | 0 | 0.015006 | 0.022834 |
| SRM4_2 | 0.017315 | 0.006637 | 0.003355 | 0.002309 | 0 | 0.002309 | 0.002309 | 0.025143 | 0.003355 | 0.006637 |
| SRM4_3 | 0.015006 | 0.004329 | 0.001046 | 0 | 0.015006 | 0.004329 | 0.001046 | 0 | 0.022834 | 0.015006 |
| SRM4_4 | 0.015006 | 0.00891 | 0.015006 | 0.022834 | 0.015006 | 0.004329 | 0 | 0.022834 | 0.001046 | 0.015006 |
| SRM5_1 | 0.015006 | 0.00891 | 0.015006 | 0.022834 | 0.015006 | 0.015006 | 0.004329 | 0.001046 | 0 | 0.015006 |
| SRM5_2 | 0.021367 | 0.006163 | 0.001489 | 0 | 0.001489 | 0 | 0.006163 | 0.001489 | 0 | 0.001489 |
| SRM5_3 | 0 | 0.002309 | 0.017315 | 0.025143 | 0.002309 | 0.017315 | 0.006637 | 0.003355 | 0.002309 | 0.003355 |
| SRM5_4 | 0 | 0.022834 | 0.001046 | 0.004329 | 0.015006 | 0.022834 | 0.00891 | 0.015006 | 0.022834 | 0.00891 |
| SRM6_1 | 0.011219 | 0 | 0.002309 | 0.017315 | 0.025143 | 0.017315 | 0.002309 | 0.025143 | 0.003355 | 0.006637 |
| SRM6_2 | 0 | 0.022834 | 0.001046 | 0.004329 | 0.015006 | 0.022834 | 0 | 0.022834 | 0.001046 | 0.004329 |
| SRM6_3 | 0 | 0.022834 | 0.001046 | 0 | 0.022834 | 0.001046 | 0.004329 | 0.00891 | 0.015006 | 0.022834 |
| SRM6_4 | 0.011219 | 0.017315 | 0.025143 | 0.017315 | 0.006637 | 0.003355 | 0 | 0.002309 | 0.017315 | 0.025143 |
| SRM6_5 | 0 | 0.002309 | 0.017315 | 0.025143 | 0.017315 | 0 | 0.002309 | 0.025143 | 0.003355 | 0.006637 |
| SRM6_6 | 0 | 0.002309 | 0 | 0.002309 | 0.017315 | 0.025143 | 0 | 0.002309 | 0.017315 | 0.025143 |
| SRM7_1 | 0 | 0 | 0.002309 | 0.017315 | 0.025143 | 0.011219 | 0 | 0.002309 | 0.017315 | 0.025143 |
| SRM7_2 | 0 | 0.002309 | 0.017315 | 0.025143 | 0.017315 | 0.025143 | 0 | 0.002309 | 0.017315 | 0.025143 |
| SRM7_3 | 0 | 0.002309 | 0.017315 | 0.025143 | 0.017315 | 0.025143 | 0.017315 | 0.006637 | 0.003355 | 0.002309 |
| SRM7_4 | 0 | 0.002309 | 0.017315 | 0.002309 | 0.025143 | 0.003355 | 0.006637 | 0.002309 | 0.017315 | 0.025143 |
| SRM7_5 | 0 | 0.022834 | 0.001046 | 0.004329 | 0.015006 | 0.022834 | 0.015006 | 0.004329 | 0.001046 | 0 |
| SRM7_6 | 0.00891 | 0.015006 | 0 | 0.022834 | 0.001046 | 0.004329 | 0 | 0.022834 | 0.001046 | 0.004329 |
| SRM8_1 | 0.011219 | 0.017315 | 0.025143 | 0 | 0.002309 | 0.017315 | 0 | 0.002309 | 0.017315 | 0.025143 |
| SRM8_2 | 0 | 0.002309 | 0.017315 | 0.025143 | 0.002309 | 0.006637 | 0 | 0.002309 | 0.017315 | 0.025143 |
| SRM8_3 | 0.011219 | 0.017315 | 0 | 0.002309 | 0.017315 | 0.025143 | 0.017315 | 0.006637 | 0.003355 | 0.002309 |
| SRM8_4 | 0 | 0.022834 | 0.001046 | 0.004329 | 0.015006 | 0.022834 | 0 | 0.022834 | 0.001046 | 0.004329 |
| SRM8_5 | 0 | 0.002309 | 0.017315 | 0.025143 | 0.025143 | 0 | 0.002309 | 0.017315 | 0.025143 | 0.002309 |
| SRM8_6 | 0.009148 | 0.015407 | 0.009148 | 0.015407 | 0.004444 | 0.001074 | 0 | 0.009148 | 0.015407 | 0.023444 |
